# SERS biosensors for liquid biopsy towards cancer diagnosis by detection of various circulating biomarkers: current progress and perspectives

**DOI:** 10.1186/s40580-024-00428-3

**Published:** 2024-05-29

**Authors:** Nana Lyu, Amin Hassanzadeh-Barforoushi, Laura M. Rey Gomez, Wei Zhang, Yuling Wang

**Affiliations:** School of Natural Sciences, Macquarie University, Sydney, NSW 2109 Australia

## Abstract

Liquid biopsy has emerged as a promising non-invasive strategy for cancer diagnosis, enabling the detection of various circulating biomarkers, including circulating tumor cells (CTCs), circulating tumor nucleic acids (ctNAs), circulating tumor-derived small extracellular vesicles (sEVs), and circulating proteins. Surface-enhanced Raman scattering (SERS) biosensors have revolutionized liquid biopsy by offering sensitive and specific detection methodologies for these biomarkers. This review comprehensively examines the application of SERS-based biosensors for identification and analysis of various circulating biomarkers including CTCs, ctNAs, sEVs and proteins in liquid biopsy for cancer diagnosis. The discussion encompasses a diverse range of SERS biosensor platforms, including label-free SERS assay, magnetic bead-based SERS assay, microfluidic device-based SERS system, and paper-based SERS assay, each demonstrating unique capabilities in enhancing the sensitivity and specificity for detection of liquid biopsy cancer biomarkers. This review critically assesses the strengths, limitations, and future directions of SERS biosensors in liquid biopsy for cancer diagnosis.

## Introduction

Cancer, a multifaceted heterogeneous disease, demands precise diagnostic methodologies to guide effective treatment strategies. Conventional diagnostic methods, while valuable, often require invasive procedures and are not favorable for multiple sampling to reflect the dynamic changes of tumor progression. Liquid biopsy, by contrast, harnesses the unique biomolecular signatures shed by tumors into body fluids, providing real-time, minimally invasive insights into the tumor’s molecular landscape [1–3]. This transformative approach involves the analysis of circulating tumor cells (CTCs), circulating tumor nucleic acids (ctNAs), circulating small extracellular vesicles (sEVs), and circulating proteins as biomarkers, offering insights into tumor dynamics and aiding in personalized therapeutic interventions.

CTCs are cancer cells that have detached from a primary tumor and intravasated into the peripheral bloodstream during the process of cancer metastasis [4, 5]. CTCs were first discovered in the blood of a man with metastatic cancer by Thomas Ashworth in 1869 [6, 7]. However, CTCs have been increasingly studied only since the mid-1990s, with the development of new isolation and detection techniques [8]. The CellSearch System (Janssen Diagnostics) is the first FDA-approved system for clinical CTCs detection in breast, colorectal, and prostate cancer patients [9]. The prognostic value of CTCs has also been demonstrated in patients with bladder, head and neck, and pancreatic cancer [10]. The detection of CTCs poses significant challenges due to multiple factors, including *i)* extraordinary rarity with approximately 1 to 100 CTCs per milliliter of blood, making their isolation and detection among 5 billion erythrocytes and 10 million leukocytes a daunting task [8, 11, 12]; *ii)* heterogeneity of CTCs exhibiting significant variations in surface marker expression, complicating the identification and characterization of CTCs [13, 14]. ctNAs include circulating tumor DNA (ctDNA) and microRNA (miRNA). ctDNAs are released from tumor cells under apoptosis, necrosis, or active release thus reflecting molecular, phenotypic or genetic changes in the tumor tissue [15–17]. miRNAs are non-coding small RNA molecules and are known for their role in promoting tumor progression and metastasis through inhibition of tumor suppressor genes as well as the genes engaged in cell apoptosis and differentiation [18]. Since ctNAs may reflect systemic disease and are more abundant than CTCs, the analysis of ctNAs could serve as a better measure of tumor burden and heterogeneity with higher sensitivity and specificity than analysis of solid tumors and CTCs [15]. sEVs, also called exosomes, are nanoscale phospholipid bilayer membrane vesicles released from cells with diameter < 200 nm [19, 20]. Due to their diverse origin (secreted by almost all types of cells) and excellent stability in the body fluid, cancer-derived sEVs have emerged as potential non-invasive biomarkers in liquid biopsy for the diagnosis and treatment of diseases [21, 22]. Circulating proteins from serum or plasma can be used as non-invasive biomarkers for cancer diagnosis, and the changes in protein expression levels and protein structures may indicate genomic mutations and reflect disease progression [23]. The detection of circulating proteins has been used in various ways, such as screening, diagnosis, monitoring treatment response, and detecting recurrence [24, 25].

The effective utilization of liquid biopsy biomarkers relies on sensitive and specific detection methodologies, propelling the advancement of surface-enhanced Raman scattering (SERS) biosensors. SERS is a powerful spectroscopic technique that enhances the Raman scattering signal of molecules adsorbed on or near nanostructured metal surfaces [26]. The enhancement is predominantly attributed to the localized surface plasmon resonance (LSPR) effect, which occurs when noble metal nanoparticles, such as gold or silver, interact with incident light [27]. SERS assays have aroused considerable attention due to their unparalleled sensitivity and specificity in detecting molecular fingerprints of various cancer-associated biomarkers. The advantages of SERS-based biosensors include *i)* extraordinary sensitivity, often allowing for the detection of molecules at extremely low concentrations [28]; *ii)* high specificity via functionalization of SERS substrates with specific ligands, antibodies, or aptamers, ensuring selective binding to target molecules [29]. This specificity is crucial for accurately identifying and distinguishing between different analytes, making SERS well-suited for biosensing applications; *iii)* high multiplexing capability, allowing for the simultaneous detection of multiple analytes in a single experiment [14, 30]. By using different SERS substrates functionalized with specific recognition elements, multiplexed analysis is achievable, providing comprehensive information in a single measurement; *iv)* non-invasive sampling, allowing for the detection of biomarkers without the need for invasive procedures; and *v)* real-time and rapid analysis, making it suitable for dynamic processes and time-sensitive applications. This feature is valuable in fields such as pharmacology, environmental monitoring, and point-of-care diagnostics [9, 31, 32]. Therefore, SERS application in liquid biopsy holds promise for revolutionizing cancer diagnostics by offering rapid, multiplexed, and non-invasive detection capabilities.

This review thus aims to comprehensively explore the recent developments and applications of SERS biosensors in liquid biopsy for cancer diagnosis. Four distinct types of SERS assay, including label-free, magnetic bead (pull-down), microfluidic device, and paper-based assays, are discussed in detail. Each type of assay exhibits unique attributes, ranging from enhanced capture efficiency to portable diagnostic capabilities, contributing to the diversification and optimization of liquid biopsy methodologies. We endeavor to elucidate the significance of liquid biopsy in cancer diagnosis and treatment management, outline the existing methodologies employed in liquid biopsy, explain the rationale behind the utilization of SERS assays, and critically analyze the recent advancements in SERS biosensors. Moreover, the specific focus is on delineating the distinct attributes and potential clinical implications of the four types of SERS assay in the realm of liquid biopsy for cancer diagnosis. By integrating the diverse facets of liquid biopsy, SERS technology, and innovative assay designs, this review aims to offer insights into the evolving landscape of cancer diagnostics and the transformative potential of SERS biosensors in enhancing precision medicine.

## Label-free SERS assay

**Fig. 1:**
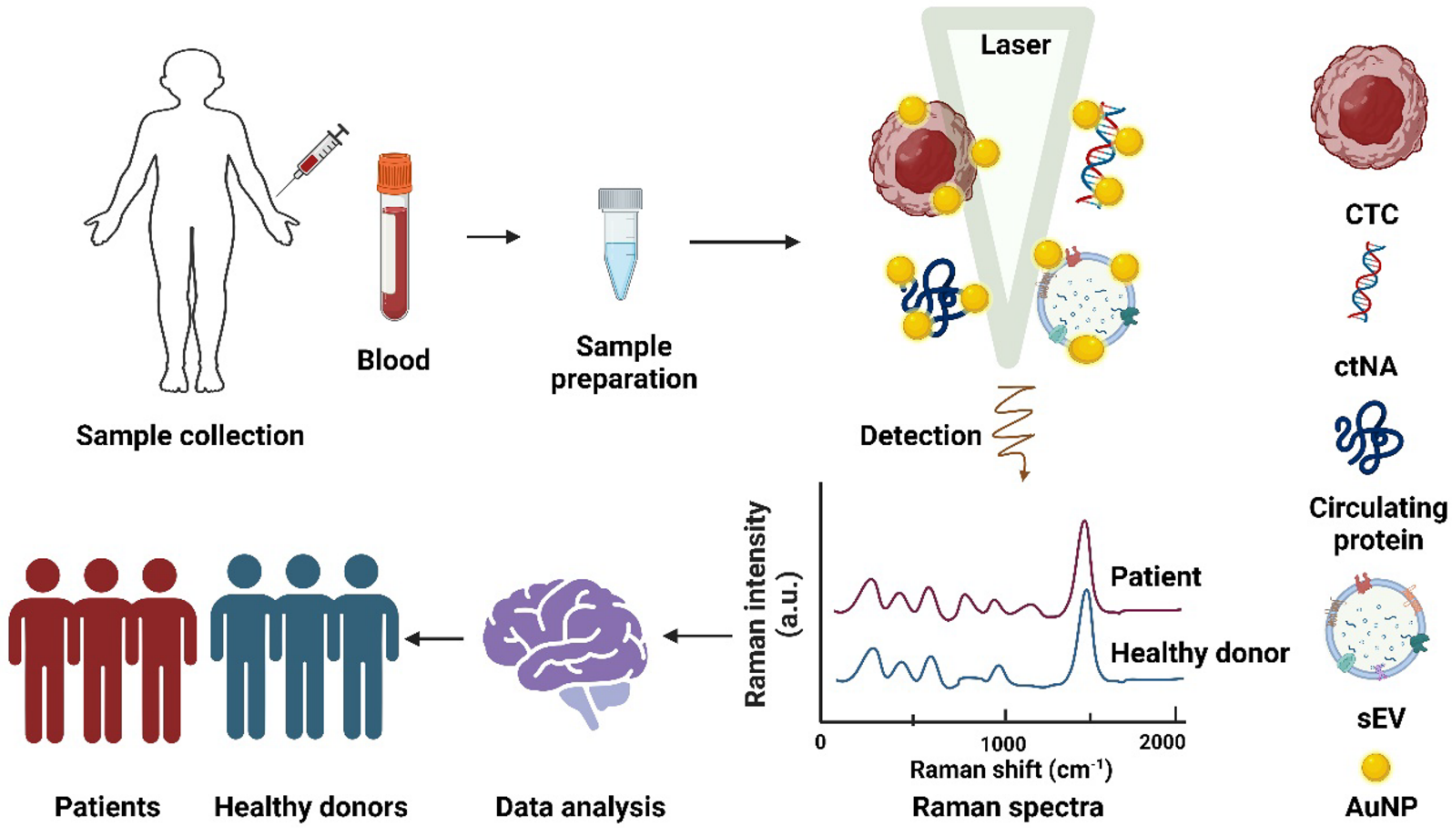
Scheme of label-free SERS assay for liquid biopsy detection. Analyte including CTCs, ctNAs, sEVs and proteins attached to the SERS substrate (such as AuNP) to generate inherent Raman spectra of the analyte, which could be analyzed by machine learning algorithms for cancer detection

Label-free SERS assay (also called direct-SERS assay) utilizes the intrinsic Raman spectrum of the analyte rather than labelling the analyte with Raman molecule to discern disease-associated biomarkers [33]. Figure 1 illustrates the working scheme of label-free SERS assay in liquid biopsy for cancer diagnosis. Noble metal nanoparticles, particularly gold nanoparticles (AuNPs) and silver nanoparticles (AgNPs), are frequently utilized as SERS substrates due to their strong plasmonic properties [34]. Spherical AuNPs display a robust plasmon resonance within the visible to near-infrared (NIR) range, typically around 520 − 550 nm [35]. This resonance makes Au an excellent choice for applications that necessitate excitation at visible wavelengths. Other materials, such as copper, aluminum, and lead, also exhibit plasmon resonance within the visible to NIR region and can be used as SERS substrate [36]. The morphology of the SERS substrate also holds a crucial role in SERS enhancement. The wavelength of the localized surface plasmon resonance for metal can be tuned by modifying the size, shape, and the dielectric layer of the nanoparticles [9]. The analyte in liquid biopsy sample can be attached to SERS substrate through physical adsorption and chemical binding. Physical adsorption relies on van der Waals forces, electrostatic attractions, or hydrophobic interactions for adhesion of analyte to the substrate [37]. Alternatively, chemical binding, such as the formation of covalent bonds, can be employed to create a more stable and specific interaction between the SERS substrate and the target [37].

In label-free SERS assay, SERS spectra of analytes provides rich fingerprint information of the analytes, indicating their molecular composition and chemical structure. However, identification of the difference in Raman spectra from complex matrix is challenging due to the potential overlapping peaks, baseline noise or intermolecular interactions. Thus, methodologies for signal analysis with more precision are highly demanded, where machine learning emerges as a promising solution. A number of algorithms, including principal component analysis (PCA), support vector machines (SVMs), convolutional neural networks (CNNs), distributed arithmetic (DA), quadratic discriminant analysis (QDA), linear discriminant analysis (LDA), partial least squares-discriminant analysis (PLS-DA), artificial neural networks (ANNs), and random forests (RFs), have been successfully employed to analyze Raman spectra [38]. When combined with an artificial intelligence (AI) technique, SERS allows for analysis of samples with enhanced accuracy, effectively addressing the limitations imposed by complex data. By leveraging advanced data analysis models, Raman spectra from healthy donors and patients can be accurately distinguished. This integration of AI and SERS offers a powerful tool for accurate and efficient diagnosis, further enhancing the capabilities of liquid biopsy in clinical applications.

### CTCs

CTCs contain a complex repertoire of biomolecules, including polysaccharides, proteins, and nucleic acids. Given its exceptional sensitivity, SERS presents a powerful analytical tool for the label-free identification of CTCs. However, accurate identification of CTCs solely through label-free SERS spectra is challenging, as the spectra of CTCs typically exhibit highly overlapping spectral features originating from diverse biomolecules within the focal area of the laser beam [33]. Therefore, appropriate algorithms capable of discerning subtle spectral variations between different cell types for accurate identification of CTCs are needed.

Zhang et al*.* presented a study that demonstrated the potential of an Ag film-based label-free SERS assay for distinguishing various cells through the utilization of a discriminant model [39]. The model was developed based on the nucleic acid characteristic peaks of various cells, including tumor cancer cell lines, CTCs derived from lung cancer patient tissues, red and white blood cells. This approach allowed for the successful discrimination of tumor cancer cells from white blood cells, as well as CTCs, by utilizing the characteristic peak intensity ratios of nucleic acid.

To simplify the procedures for CTC isolation and analysis, one study presented a one-step technique based on tailor-made membrane to isolate and enrich CTCs from blood samples [40]. SERS-active nanoparticles (Ag-Au alloy) on a polymer mat could enhance the Raman signals, enabling molecular and biochemical analysis of CTCs. SERS spectra of prostate cancer cells (PC3), cervical carcinoma (HeLa) cells, and leucocytes (representing healthy cells) showed distinctive differences in band positions and intensities of the Raman spectra, thus PCA effectively identified these key differences and achieves accurate classification of cell types, demonstrating the potential for efficient cancer discrimination.

In an alternative methodology, Niciński et al*.* leveraged the advantages of automation and high-throughput capabilities provided by microfluidic devices to isolate and detect CTCs in human blood [41]. The microfluidic chip enabled efficient size-based inertial separation of CTCs, and the SERS-active substrate (AgNPs coated with silica shell) facilitated label-free detection and molecular identification of isolated cells. SERS analysis discerned significant differences in molecular composition between cancer cells (HeLa, Caki-1) and blood cells using PCA. This innovative approach simplifies the detection of CTCs, improving accuracy without invasiveness or cell damage.

### ctNAs

Label-free SERS technology for ctNAs detection facilitates the direct acquisition of Raman signals from nucleic acids, encompassing both nucleobases and the phosphate/sugar backbone [42]. This capability enables direct identification of base changes and conformational alterations within nucleic acid structures. Label-free SERS has demonstrated successful detection of single bases, point mutations, base methylations, and structural modifications, indicating the remarkable potential of label-free SERS as a sensitive and rapid method for ctNA detection [43, 44].

For instance, Liu et al*.* successfully created positively charged Au–Ag alloy nanostars to perform label-free SERS detection of DNA mutations without the need for Raman molecule labelling. The nanostars were utilized to distinguish between wild-type (WT) and BRAF V600E mutant genomic DNA using statistical analysis methods known as PCA-LDA [45]. This approach allowed for the analysis of whole genome DNA lysed from cells. Notably, the method provided a comprehensive DNA fingerprint with reduced analysis time while achieving a low detection limit of 100 copies, which was comparable to quantitative PCR (qPCR) but ten times more sensitive than traditional gel electrophoresis.

In another study, Lin et al*.* demonstrated the label-free SERS method for sensitive quantification of minor changes in DNA molecules at the single nucleobase level [42]. The proposed method detected circulating DNA in blood and achieved the diagnostic sensitivity of 83.3% and specificity of 82.5% for differentiating nasopharyngeal cancer (NPC) patients from normal subjects. This proof-of-concept study demonstrated the promising potential of the method for sensitive NPC detection based on liquid biopsy.

Label-free SERS can also be used for RNA detection. For example, a label-free SERS assay, coupled with a duplex-specific nuclease (DSN) signal amplification strategy, has been developed for the sensitive and quantitative analysis of miRNA-21 [46]. This approach involved utilizing magnetic beads functionalized with excess capture DNA to hybridize with the target miRNA-21 and iodide-modified Ag nanoparticles (AgINPs) for SERS detection. This method indicated excellent performance for miRNA-21 detection at a lower detection limit of 42 aM. Furthermore, this strategy exhibited effective base discrimination capability and was successfully applied to monitor the expression levels of miRNA-21 in different cancer cell lines and human serum.

### Circulating tumor-derived sEVs

Label-free SERS, which investigates signal patterns that can originate from either unidentified or non-interesting substances, enables the analysis of sEVs that are challenging to differentiate using other analytical methods that target a unique marker. Additionally, Raman spectrum of sEVs contains abundant information regarding the chemical structure, thereby offering insights into the molecular composition of sEVs.

For example, one research introduced an artificial intelligence-based SERS strategy for label-free spectral analysis of serum sEVs [47]. The deep learning algorithm training by using SERS spectra from cancer cell-derived sEVs demonstrated 100% prediction accuracy for patients with different breast cancer subtypes and did not undergo surgery. Moreover, when combined with similarity analysis through PCA, the approach could effectively evaluate the surgical outcomes for distinct molecular subtypes of breast cancer.

To investigate the clinical application of label-free SERS for sEV detection, a large cohort of patient samples with different cancer types were investigated using AgNPs as SERS susbtrate [48]. SERS spectra of serum and serum-derived sEVs from 32 patients with prostate cancer (PCa), 33 patients with renal cell cancer (RCC), 30 patients with bladder cancer (BCa), and 35 healthy controls (HCs) were obtained using label-free SERS assay, yielding 650 and 1206 spectra, respectively. The serum SERS-based CNN models showed testing accuracies of 73.0%, 71.1%, and 69.2% in diagnosing PCa, RCC, and BCa, respectively. These results showed the superior diagnostic potential of deep learning-based SERS analysis of sEVs, providing a novel and effective tool for the diagnosis of urologic cancer, outperforming serum-based SERS analysis.

Additionally, Shin et al*.* utilized deep learning-based SERS assay of sEVs, and achieved an accurate diagnosis of early-stage lung cancer [49]. Analyzing 43 patients, including those at stages I and II, the deep learning model predicted a significant similarity between plasma sEVs and lung cancer cells-derived sEVs in a substantial proportion of patients. This similarity correlated with cancer progression, and notably, the model predicted lung cancer with a high area under the curve (AUC) of 0.912 for the entire cohort and 0.910 for stage I patients. These findings underscored the immense potential of combining sEVs analysis and deep learning for early-stage liquid biopsy of lung cancer.

More recently, Liu et al*.* applied an Au nanopyramid array as the SERS substrate which could boost a high density of hot spots with SERS enhancement factor over 10^10^ to obtain composition information from Raman-active bonds inside sEVs [50]. A machine learning-based spectral feature analysis algorithm was developed to distinguish cancer-derived sEVs from non-cancer sub-populations objectively. The algorithm demonstrated prediction accuracies of 90%, 85%, and 72% in tissue, blood, and saliva, respectively. A cross-validation method was conducted to evaluate the performance of a diagnostic or prognostic model and assess the clinical potential, where excellent predictive accuracy was indicated by the high AUC in ROC analysis. Furthermore, this study proposed a way to trace the biogenesis pathways of patient-specific sEVs from tissue to blood to saliva by comparing the SERS fingerprints of individual vesicles.

### Circulating tumor-related proteins

Label-free SERS stands as a prominent approach for the detection and in-depth characterization of proteins. This technique affords comprehensive structural insights into proteins by revealing vibrational details from crucial elements such as amide groups, amino acids, and protein cofactors, including heme and flavins, under physiological conditions. Notably, label-free SERS is not limited by the protein molecular mass or solubility, thus offering distinct advantages over traditional protein analysis methods such as electrophoresis, enzyme-linked immunosorbent assay (ELISA), and western blots.

For instance, Chaloupková et al*.* developed an analytical method for the parallel analysis of prostate-specific antigen (PSA) and free PSA in whole human blood using magnetically assisted (MA)-SERS [51]. This method was based on magnetic Fe_3_O_4_@Ag nanocomposite functionalized with anti-PSA antibody. It could distinguish between the levels of PSA and free PSA within a single analytical run with LOD of 0.62 ng/ml and 0.49 ng/ml for PSA and free PSA, respectively.

Additionally, Liu et al*.* used label-free SERS technology combined with AgNPs to measure and analyze peripheral serum protein samples from patients with breast cancer, pre- and postoperatively, and from normal subjects [52]. Significant differences in the serum protein’s SERS spectra among the three groups were detected due to the changes in certain biochemical compositions related to breast cancer transformation. Using PCA-LDA, the authors achieved diagnostic sensitivities of 96.7%, 53.3%, and 100% for pre-surgery versus post-surgery, post-surgery versus normal, and pre-surgery versus normal, respectively.

In a short summary, the development of a label-free SERS assay for liquid biopsy detection represents a promising potential toward clinical translation. This technology enables the sensitive and specific detection of biomarkers directly from complex biological samples, eliminating the need for labelling Raman molecules. The key to its efficacy lies in the utilization of nanomaterials, such as Au and Ag nanoparticles, which amplify the Raman scattering signal of the target analyte. By capitalizing on surface plasmon resonance, Raman signal is significantly boosted, thereby facilitating sensitive detection of analytes at low concentrations. Furthermore, advancements in data analysis models, including machine learning algorithms, have contributed to the improvement of label-free SERS assays. Together with high-resolution Raman spectrometers and sophisticated algorithms for signal extraction and background correction, these advancements have enabled more accurate and reliable measurements. To provide a comprehensive overview of label-free SERS assays for the detection of circulating biomarkers in recent five years, Table 1 summarizes the key information regarding assay sensitivity, SERS substrate, sample sources, data analysis methods, assay advantages, clinical sample details, and associated disease types. This table serves as a valuable resource for understanding the current state of label-free based SERS detection techniques for circulating tumor biomarkers.

**Table 1:** Label-free SERS assay for the detection of circulating tumor biomarkers

Target	Sensitivity	SERS substrate	Sample sources	Analysis method	Merits	Clinical samples	Disease	Ref.
CTCs	Single cell	ZnO-based 3D semiconductor quantum probe	Cell lines	PCA	Quantum probes were used for multiple, simultaneous SERS detection of cells, as well as biomolecular detection up to single-cell-level	N/A	N/A	[53]
CTCs	N/A	Ag film	Cell lines, blood cells	N/A	Label-free SERS technology could effectively identify blood cells and tumor cells	N/A	Lung cancer	[39]
CTCs	N/A	Ag film	Cell lines, blood cells	N/A	SCLC cell lines and their clinical cell samples were completely distinguished by the intensity ratio of the characteristic peak to the subtracted background peak	N/A	Small cell lung cancer (SCLC)	[54]
CTCs	N/A	SERS substrates with a thin layer of Ag/Au alloy	Spiked Blood	PCA	The studied cell types were classified with an accuracy of 95% in 2D PCA to 98% in 3D PCA	N/A	Prostate cancer, cervical carcinoma	[40]
CTCs	N/A	Ag@SiO_2_	Cell lines, blood cells	PCA	The established statistical model achieved diagnostic accuracy up to 89% for differentiation of blood cells, Caki-1 and HeLa cells	N/A	Renal cell carcinoma, cervical carcinoma leukemia	[41]
CTCs	20 cells/mL	Ag coated silicon	Cell lines, spiked plasma	N/A	The chip could trap the CTCs on the surface of SERS platform and LOD was down to 20 cells/mL	N/A	Breast cancer	[55]
DNA	Single nucleobase	AgNPs	Blood	PCA-LDA	The SERS method combined with PCA-LDA was applied in real blood circulating DNA detection for the first time to differentiate the NPC from the normal group	Non-cancer volunteers (*n* = 120) and NPC patients (*n* = 120)	Nasopharyngeal cancer (NPC)	[42]
DNA	N/A	Au@Ag NRs	Cell lines and CRC patients	CLS-LDA	System performance is verified by classifying cancer patient samples with an accuracy above 90%	8 CRC patients	Colorectal cancer (CRC)	[56]
DNA	Single-molecule	Graphite substrate-based quantum semiconductor	Cell lines	PCA	The method was able to detect divergences in genomic DNA of cancerous and noncancerous cells and trace the expression of two genes markers	N/A	Breast cancer, pancreatic cancer, and lung cancer	[57]
RNA	42 aM	AgINPs	Cell lines, serum	N/A	Expression levels of miR-21 in different number of cancer cells and human serum (normal subjects and patients) were quantitated	N/A	Cervical, lung, breast, liver cancer	[46]
DNA	1 pg/μL	Photo-etched GaN covered with Au layer	Tissue, plasma	N/A	The GaN substrates modified with thiolated ssDNA was successfully used for analysis of clinical samples	17 clinical samples	Thyroid, melanoma	[58]
DNA	100 copies	Au/Ag nanostars	Cell lines, plasma	PCA-LDA	The method was able to differentiate mutant DNA from whole genome DNA lysed from cell lines, cell-free DNA collected from cell culture media and plasma samples	N/A	CRC	[45]
DNA	10^4^ copies	AgNPs	Cell lines, patient urine samples	N/A	Elevated T2:ERG and PCA3 gene levels were positively associated with high-risk PCa on biopsy	N = 73	Prostate cancer (PCa)	[59]
sEVs	N/A	Au nanopyramids	Cell lines, serum	PCA	Identification of unique signatures of sEVs from different sources	Healthy human serum	Lung cancer	[60]
sEVs	N/A	Au nanostar	Cell lines, serum	ANN	Distinguish sEVs from different pathological origins with subtle variations of spectral features and overcome issues of complicated spectral patterns	34 BCa patient samples	Breast cancer (BCa)	[47]
sEVs	N/A	AgNPs	Serum	CNN	CNN models of sEVs spectra revealed high testing accuracies of 79.3%, 78.7% and 74.2% in diagnosis of PCa, RCC and BCa	32 PCa patients, 33 RCC patients, and 30 BCa patients	PCa, renal cell cancer (RCC), BCa	[48]
sEVs	N/A	AuNP coated plate	Cell lines, plasma	Residual neural network-based deep learning model	The model predicted lung cancer with AUC of 0.912 for the whole cohort and stage I patients with an AUC of 0.910	43 lung cancer patients, including stage I and II	Lung cancer	[49]
sEVs	N/A	Au nanopyramid arrays	Tissue, blood, and saliva	LDA, SVM	The AUC of each ROC curve was 0.96, 0.91, and 0.65 in tissue, blood, and saliva, respectively	15 samples for each (tissue, blood, and saliva)	Gastric Cancer	[50]
sEVs	N/A	Fe_3_O_4_/Au NPs	Cell lines, serum samples	PCA	Effectively distinguished sEVs from different cell sources for cancer diagnosis and showed high sensitivity and specificity within a 95% confidence interval	Healthy mice and mice with breast cancer	BCa	[61]
sEVs	N/A	AuNPs	Cell lines, serum	Principal component differential function analysis (PC-DFA)	This method exhibited up to 87% and 90% predictive accuracy for healthy control and early pancreatic cancer individual samples, respectively	10 early pancreatic cancers	Pancreatic cancer	[62]
sEVs	N/A	3D plasmonic AuNPs nanomembranes	Cell lines, serum	LDA	The LDA model identify sEVs from three different cell lines, with 93.3% prediction accuracy for human serum exosomes	Human serum	BCa and cervical cancer	[63]
sEVs	N/A	AuNP-aggregated array chip	Plasma	CNN	The final integrated decision model showed high sensitivity and specificity while predicting tumor organ of 72% of positive patients	4943 HC and 18,108 cancer samples	Lung, breast, colon, liver, pancreas, and stomach cancer	[64]
Protein	0.49 ng/mL for free PSA	Magnetic Fe_3_O_4_@Ag	Blood	Partial least squares discriminant analysis	The method could distinguish between levels of PSA and free PSA within a single analytical run with LODs of 0.62 ng/ml for PSA and 0.49 ng/ml for free PSA, respectively	N/A	Prostate cancer	[51]
Protein	N/A	Au nanostars	Cell lines	PCA-LDA	By combining label-free SERS detection and machine learning-driven chemometric analysis, they can identify and classify the breast cancer cells with distinct HER2 expression at high accuracy	N/A	BCa	[65]
Protein	N/A	AgNPs	Serum	PCA-LDA	The authors achieved diagnostic sensitivities of 96.7%, 53.3%, and 100% for pre-surgery versus post-surgery, post-surgery versus normal, and pre-surgery versus normal, respectively, with diagnostic specificities of 96.7%, 46.7%, and 96.7%, respectively	N/A	BCa	[52]

Despite its promising potential, label-free SERS still faces challenges in its journey to clinical translation, which includes overcoming background noise and achieving reproducibility. Future research should focus on addressing these challenges to fully harness the capabilities of label-free SERS in liquid biopsy applications: *i)* overcoming background noise from other untargeted substances, which will involve significant consideration of sample preparation to minimize the background signal; *ii)* developing SERS substrates with high enhancement factors, and stability; *iii)* creating advanced data analysis tools for accurate analysis and identification of analytes from complex SERS spectra; *iv)* establishing standardized protocols and rigorous clinical validation methods to ensure reproducibility and reliability of SERS assays in clinical settings. Addressing these challenges through collaborative efforts between researchers, clinicians, and engineers will be key to unlocking the full potential of label-free SERS for liquid biopsy applications.

## Magnetic bead-based SERS assay

**Fig. 2:**
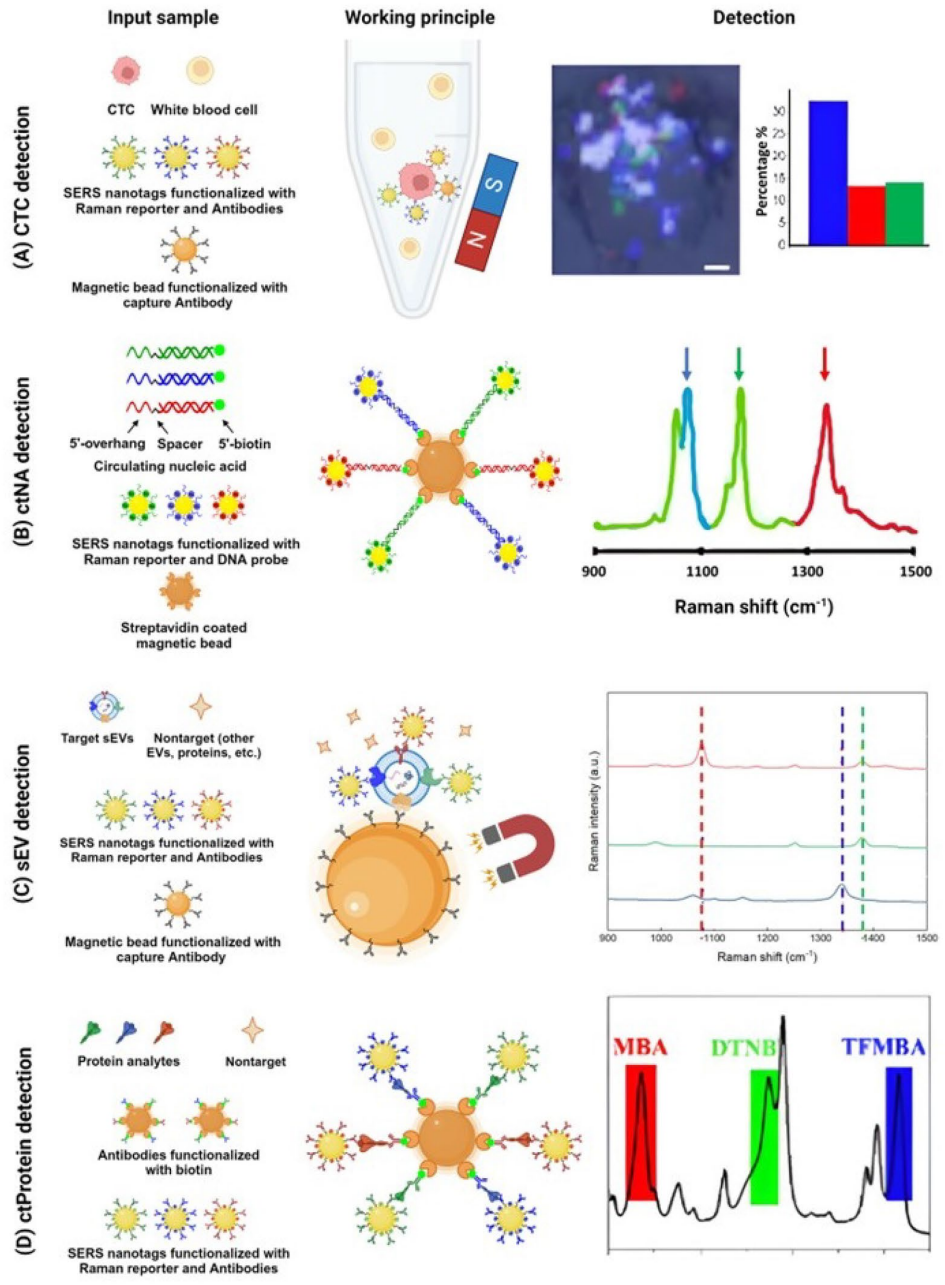
Scheme of beads-based SERS assay for detecting circulating biomarkers, where SERS nanotags are functionalized with specific ligands, e.g., antibodies, aptamers, or DNA probes for selective identification of the biomarkers. **A** CTC detection, where CTC surface markers are labelled with SERS nanotags and magnetic beads, followed by magnetic enrichment for SERS reading. Reproduced with permission [14]. Copyright 2018, Springer Nature. **B** ctNA detection, where amplicons were tagged with mutation-specific SERS nanotags and enriched using magnetic beads for SERS detection. Reproduced with permission [30]. Copyright 2016, Ivyspring International Publisher. **C** sEVs detection, where molecular phenotype profiling of sEVs were detected using SERS nanotags and capture antibody-functionalized magnetic beads. Reproduced with permission [79]. Copyright 2020, American Chemical Society. **D** Protein analytes were recognized by magnetic beads functionalized with capture antibodies and SERS nanotags for readout of SERS signal. Reproduced with permission [10.1021/acssensors.9b01211]. Copyright 2019, American Chemical Society

When combined with magnetic beads or nanoparticles, SERS creates a versatile platform for the detection of biomarkers from liquid biopsy. In the magnetic bead-based SERS assay, SERS nanotags are employed as sensors. Typically, SERS nanotags contain a SERS substrate (plasmonic active metal colloids such as gold and silver), Raman reporters adsorbed onto their surface, and conjugation with a target-specific binding molecule, e.g., antibodies, aptamers, or DNA probes for selective identification of the biomarkers [29]. The complexes of SERS nanotags/biomarkers are then recognized by magnetic beads functionalized with capture ligands to form a SERS nanotags/biomarker/magnetic bead sandwich structure for enrichment by a magnet and the following SERS measurement. The integration of magnetic bead with SERS technology enhances the sensitivity, specificity, and overall performance of liquid biopsy biomarker detection assays. This approach holds great promise for early disease diagnosis and monitoring, especially in the field of personalized medicine. This subsection aims to delve into the diverse applications, methodologies, and recent advancements in magnetic bead-based assays for detecting CTCs, ctNAs, sEVs, and proteins from liquid biopsy. The discussion will encompass various strategies involving magnetic beads functionalization, assay design, and their role in enhancing the sensitivity and specificity of cancer biomarker detection. The scheme of bead-based SERS assay is illustrated in Fig. 2.

### CTCs

Magnetic beads offer a way to selectively isolate the target CTCs from numerous other blood cells by exploiting specific interactions between the beads and the target cells. This enrichment step can improve the sensitivity and specificity of the SERS assay, enhancing the detection of CTCs amongst a diverse population of blood cells. The combination of magnetic beads with SERS-based detection was firstly reported by Sha et al. (2008), where the bead modified with anti-EpCAM antibody and SERS nanotags with anti-HER2 were used for specific recognition of breast cancer cells in whole blood. The typical sandwich structure (magnetic beads-CTC-SERS nanotags, Fig. 2A) was formed for rapid capture and detection of CTCs in whole blood with a LOD of 50 cells/mL [66].

To improve the assay sensitivity, SERS nanotags with improved signal enhancement were designed for CTCs detection. For example, Ruan et al*.* designed the triangular silver nanoprisms (AgNPR) and superparamagnetic iron oxide nanoparticles (SPION), both functionalized with folic acid for capture, enrichment, and detection of cancer cells in the blood with high sensitivity (1 cell/mL) [67]. Notably, the captured CTCs were further released via excessive free folic acid for cell expansion and phenotype identification [68].

Alternatively, modification of magnetic beads with gold or silver-coated shell can further improve the SERS signal due to their enhanced plasmonic properties. For instance, Pang et al*.* fabricated silver shell-coated magnetic nanoparticles functionalized with anti-ASGPR antibody and Au@Ag nanorods functionalized with anti-GPC3 for detection of CTCs from hepatocellular carcinoma (HCC) [69]. Wherein, a LOD of 1 cell/mL for HCC CTC in human peripheral blood samples was obtained due to the dual-enhanced SERS signals between the silver shell and the Au@Ag nanorods.

### ctNAs

PCR-based assays for detecting ctNAs mutations rely on probe-based qPCR, or targeted sequencing [70]. More recently, droplet digital PCR has been demonstrated to quantify mutant copies from limited DNA input in ctNAs [71]. While accurate, these fluorescence-based methods require expensive specialized equipment, have limited sensitivity and multiplexing capability [30]. In the magnetic bead-based SERS assay, the combination of biochemical (PCR) and physical (SERS) amplification allows the assay to detect low copies of aberrant DNA from wild type sequences, meanwhile, the magnetic beads can be easily separated from the solution using magnetic fields to simplify the sample preparation and washing steps.

Wee et al*.* reported a PCR/SERS method for multiplex detection of clinically important melanoma DNA mutations in ctDNA, by using a biotinylated reverse primer for capture by streptavidin-coated magnetic beads (SMB), and an allele-specific forward primer for direct hybridization to SERS nanotags. The presence of targeting amplicons was detected by the fingerprinting spectrum of the SERS nanotags (Fig. 2B) [30]. PCR/SERS method showed comparable sensitivity with that of droplet digital PCR (ddPCR) yet at the convenience of standard PCR, thus illustrating the great potential in sensitive detection of multiple ctDNA mutations in clinical setting. Lyu et al*.* further improved this PCR/SERS assay with a simple and specific strategy by integrating asymmetric PCR with SERS (Asy-PCR/SERS) for highly specific distinguishing of KRAS G12V (c.35G > T) and KRAS G12D (c.35G > A) locating at the same nucleotide on KRAS oncogene without the needs of complex design and optimization of allele-specific primers compared to that of PCR/SERS assay [72].

DNA probe-based method is a direct and efficient method for miRNA detection in blood samples and is suitable for both research and clinical applications. For example, Wu et al*.* have demonstrated simultaneous and sensitive detection of three hepatocellular carcinoma-related miRNA biomarkers, namely miRNA-122, miRNA-223, and miRNA-21 by using SERS nanotag and a magnetic capture substrate [28]. Other magnetic nanoparticles-based SERS platforms with elaborate design of signal amplification are also reported. For instance, He et al*.* proposed a novel “off” to “on” SERS platform combining padlock probe-based exponential rolling circle amplification strategy and magnetic Co@C/PEI/Ag SERS substrate for quantitative and sensitive detection of miRNA-155 with a LOD of 70.2 aM [73].

### Circulating tumor-derived sEVs

ELISA and western blotting are two regular approaches widely used to detect proteins from sEVs [74, 75]. However, these methods are limited by low sensitivity and complicated protocols. The immunoaffinity magnetic beads for enrichment of sEVs through their specific protein markers have been used for the point-of-care clinic diagnosis as the simple and fast isolation process [76]. The mechanism of immunomagnetic isolation protocols is using magnetic beads coated with anti-marker antibodies/aptamers to capture sEVs by recognizing the specific proteins on their surface (Fig. 2C) [77].

A variety of proteins can be targeted as biomarkers for isolation of sEVs including the tetraspanins CD9, CD63, CD81 and cancer-related markers such as EpCAM, CD24, and CA125. Antibodies can be immobilized on the surface of beads for binding with sEVs that expressed specific antigens, the immunoaffinity thus resulted in high specificity and purity for isolating a particular sEVs subtype (Fig. 2C) [78]. For example, Zhang et al*.* reported using anti-CD63 modified magnetic beads to capture and enrich pancreatic cancer-derived sEVs from different cell lines, followed with multiplex detection of three surface markers glypican-1, EpCAM, CD44v6 on captured sEVs by the three corresponding antibody labelled SERS nanotags [79]. To further demonstrate the clinical application of the proposed assay, they profiled the sEVs’ phenotypes from healthy donors and pancreatic ductal adenocarcinoma patients, providing an initial investigation of using bead-based SERS assay for pancreatic cancer diagnosis and early cancer stage prediction in the clinical setting [80].

Aptamers have been used as an alternative to antibodies with high selectivity and affinity toward protein biomarkers [81]. Numerous aptamer-based biosensors have been designed for sEVs detection [82]. Taking the advantage of particularly designed aptamers and the multiplexing ability of the SERS spectra, Wang et al*.* proposed a SERS-based method for screening and simultaneous multiple detection of sEVs using magnetic substrates and SERS probes for targeting sEVs’ proteins (CEA, PSMA, HER2) [83].

Other than the above-mentioned immunoaffinity binding between the functionalized magnetic beads and sEVs, Pang et al*.* reported a strategy to enrich sEVs through the binding of hydrophilic phosphate head of the sEVs phospholipids to the TiO_2_ shell on Fe_3_O_4_@TiO_2_ nanoparticles, where sEVs could be enriched and separated from solution within 5 min with a capture efficiency of 96.5%, and subsequent labelling with anti-PD-L1 antibody modified Au@Ag@MBA SERS nanotags for quantification with a LOD of 1 EV/μL [84]. Jiang et al*.* further reported using the same strategy for enrichment of sEVs through the affinity interaction of TiO_2_ shell on Fe_3_O_4_@TiO_2_ nanoparticles, while using locked nucleic acid (LNA)-modified Au@DTNB SERS nanotags to bind with target miRNAs inside sEVs to induce hot spot SERS signals. This is the first attempt to apply the target-triggered hot spot SERS strategy for cancer-related miRNA qualification inside sEVs, where sEVs miRNA can be determined directly in serum samples [85].

Magnetic bead-based SERS assay has proved the promise in the early-stage screening of cancers. With the further improved sensitivity and repeatability, the SERS-based method will be a useful strategy in the diagnosis and therapeutics of cancers. However, due to the high cost of affinity-based assay, this method is only applicable for a small volume of samples and might not be suitable for processing large volumes of samples efficiently.

### Circulating tumor-related protein

The magnetic-assisted sandwich strategy is the most used detection strategy for protein biomarkers. Generally, magnetic bead/nanoparticle modified with antibodies or aptamers are used to capture the target proteins, followed by the binding/detecting of SERS nanotags in the sample solution (Fig. 2D). Cheng et al*.* reported a bead-based SERS assay for simultaneous detection of dual PSA makers namely free PSA (f-PSA) and complexed PSA (c-PSA) from prostate cancer patients by using the total PSA (t-PSA) antibody-conjugated magnetic beads as capture substrates and two different types of antibody-conjugated SERS nanotags as detection probe [86].

Other than antibodies, aptamer could also be used for recognizing the target proteins. For example, Hu et al*.* have demonstrated the use of aptamer labelled SERS nanotags (Raman reporter labelled Au nano-bridged nanogap particles, Au NNPs) and magnetic capture substrate (Ag-coated Fe_3_O_4_-Au nanoparticles, Ag MNPs) for specific recognition of C-reactive protein (CRP). The method exhibits excellent selectivity and specificity for CRP under the interference of other proteins and displays high accuracy in the detection of human serum samples [87].

In a short summary, the high sensitivity and specificity of magnetic bead-based SERS assay allow for the detection of trace amounts of biomarkers in liquid biopsy, which is crucial in early cancer detection. Meanwhile, magnetic beads can be easily separated from the solution using magnetic fields, which not only simplifies the sample preparation and washing steps, but also reduces the background noise and interferences. Moreover, magnetic bead-based SERS assay is compatible with various sample matrices and can be used for both research and clinical application; this flexibility makes it a versatile tool for liquid biopsy detection. Table 2 summarized magnetic bead-based SERS assay in the detection of liquid biopsy, listed with typical reports in terms of sensitivity, specificity and multiplexity published in the last 5 years. With further optimization and improvement, the method may achieve higher selectivity, and reproducibility. In addition, more studies are needed to validate the method in clinical settings and to evaluate its diagnostic accuracy in larger patient cohorts.

**Table 2:** Detection of circulating biomarkers with magnetic bead-based assay

Target	Sample type/sources	SERS substrates	Biomarkers	Sensitivity (LOD)	Merits	Demerits	Clinical samples	Ref.
CTCs	PBMCs extraction and CD45^+^ depletion	Gold-silver alloy nanoboxes	PD-L1, MHC-1, MHC-II, MCSP	10 cells/7.5 mL of blood	Simultaneous profiling of multiple biomarkers	CTC isolation is required	A cohort of 14 late-stage melanoma patients	[88]
CTCs	Treatment of blood with separation medium to collect monocyte cells	Fe_3_O_4_@SiO_2_@Au nanoparticles	EpCAM	1 cell/mL in PBS	Good linearity between ratiometric SERS signal and cells concentration	Complexity of DNA walker design and limited multiplexing	4 healthy donors and 6 breast cancer patients	[89]
CTCs	Whole blood without pretreatment	Au-rGO@anti-ErbB2 nanotag	ErbB2	5 cells/mL in blood	Isolation and detection of CTCs with lab-on-a-filter system	Long-term performance and durability of the device is a concern	N/A	[90]
CTCs	Treatment of blood with lymphocyte isolation medium to collect monocyte cells	SPION-PEI@AuNPs-MBA-rBSA-FA	Folate receptor	1 cell/mL of blood	Quantitative measurement of CTCs and release of CTCs for molecular phenotyping	Single-plex detection only for CTCs with folate receptor expression	2 first-stage clinical patients with cervical cancer	[68]
CTCs	PBMCs extraction and CD45^+^ depletion	AuNPs with Raman reporters and antibodies	MCSP, MCAM, ErbB3, and LNGFR	10 cells/10 mL of blood	Simultaneous profiling of multiple biomarkers	CTC isolation is required	10 stage-IV melanoma patients	[14]
CTCs	Whole blood without pretreatment	Core–shell plasmonic nanoparticles (Au@Ag@DTNB)	GPC3	1 cell/mL in blood	A supplement for detecting EpCAM-negative CTCs	Single marker detection is not suitable for identifying heterogeneous CTCs	8 hepatocellular carcinoma patients, 3 breast cancer controls and 3 healthy controls	[69]
CTCs	Whole blood without separation	Triangular silver nanoprisms	Folate receptor	1 cell/mL in blood	Further release of captured CTCs for cell expansion and phenotype identification	Single marker detection is not suitable for identifying heterogeneous CTCs	N/A	[67]
CTCs	Whole blood without separation	AuNPs coated with Raman reporters and antibodies	KDED2a‑3 aptamer binding site	100 cells/0.5 mL of blood	Accurate identification of CTCs from whole blood sample	Specificity and sensitivity of aptamer binding may not always be as high as that of antibodies	N/A	[91]
CTCs	Whole blood without separation	Silver-coated gold nanorods (AuNR/Ag)	Keratin 18, IGF-1, CD44, EpCAM	10 cells/mL in blood	Multiplex targeting for improving multispectral imaging of single CTC	Complex synthesis of silver-gold nanorods with specific surface properties for CTC detection	N/A	[92]
ctDNA	N/A	AuNPs coated with Raman reporters and DNA probes	KRAS G12V, KRAS G12D	0.1% mutant alleles	Distinguish KRAS G12V and G12D that occur at the same nucleotide location	Detection of a single SNP may not be suitable for broader genomic analysis or disease screening	N/A	[72]
ctDNA	Isolation of ctDNA from 1 mL of plasma	AuNPs coated with Raman reporters and DNA probes	KRAS G12V, KRAS G13D, and BRAF V600E	0.1% mutant alleles	With ddPCR-like sensitivity yet at the convenience of standard PCR	Requires specialized expertise to optimize allele-specific primers	9 blood samples from advanced CRC patients	[93]
ctDNA	Isolation of ctDNA from less than 1.5 mL of plasma	Ag colloids	BRAF V600E, KRAS G12C, KRAS G12D, KRAS G12V, KRAS G13D, and PIK3CA E542K	5.15 × 10^−11^ M	PCR-SERS method is multiplexed and sensitive to detect mutations in blood sample	Mutation sequences should be known beforehand for designing probes	Blood samples of 49 colorectal cancer patients	[94]
ctDNA	Isolation of ctDNA from less than 1.5 mL of plasma	Ag colloids	EGFR mutations at exons 19 and 21	5.97 × 10^−11^ M for EGFR 19, 9.24 × 10^−12^ M for EGFR 21	PCR-SERS is non-invasive assay for detecting mutations from plasma	MAS-PCR is designed to detect specific mutations, and it may not cover the entire mutational spectrum of EGFR	Plasma of 48 patients with non-small cell lung cancer	[95]
ctDNA	Isolation of ctDNA from 1 mL of plasma	AuNPs coated with Raman reporters and DNA probes	BRAF V600E, c-Kit L576P, NRAS Q61K	0.1% (10 copies)	Multiplex detection of clinically important DNA mutations from limited volume of blood	Requires specialized expertise to optimize allele-specific primers	Plasma of 5 melanoma patients	[30]
miRNA	Centrifugation of blood to obtain serum	Raman reporter-labeled DNA-AuNPs	miRNA-122, miRNA-223, and miRNA-21	349 aM for miRNA-122, 374 aM for miRNA-223, and 311 aM for miRNA-21	Simultaneously sensitive and specific detection of multiple miRNAs	Multiplex microRNA detection may not capture the full spectrum of cancer heterogeneity	Human blood samples obtained from HCC patients (*n* = 92)	[28]
miRNA	N/A	Au@Ag core–shell nanoparticles	miRNA-21	0.084 fM	Good sensitivity for miRNA detection	No real blood samples from patients are analyzed	N/A	[96]
miRNA	N/A	Co@C/PEI/Ag SERS substrate	miRNA-155	70.2 aM	A novel “off” to “on” SERS platform for miRNA 155 detection	No real blood samples from patients are analyzed	N/A	[73]
miRNA	70 − 80 μL of RNA extracted from mice blood	Au shell nanoparticle	miR-122	8 fM	Detection of disease-related exosomal miRNA	Limited mice samples are analyzed	Tail blood from seven C57BL/6 mice	[97]
sEVs	30 μL of filtered serum sample	Au@Ag core–shell bimetallic nanostructure	Prostate-specific membrane antigen	19 particles/μL	Results could be obtained within 40 min with a detection limit of 19 particles/μL	Obtaining aptamers that exhibit high affinity and specificity for exosomes can be challenging	Serum samples from 10 prostate cancer patients and 9 healthy donors	[98]
sEVs	100 μL of sEVs (10^6^/μL)	Gold nanoparticles conjugated with Raman reporters and antibodies	EpCAM, CA125, and CD24	1.5 × 10^5^/μL	Multiplex detection of biomarkers shows high specificity for detecting overexpressed sEVs	Detection sensitivity is not as high as other reported methods	N/A	[99]
sEVs	10 μL of pre-treated plasma	Gold nanoparticles conjugated with Raman reporters and antibodies	Glypican 1, EpCAM and CD44V6	2.3 × 10^3^ /μL	Multiplex detection of surface proteins on plasma-derived sEVs shows high accuracy for PDAC diagnosis	Quantifying the number of exosomes based on SERS signals can be challenging	Plasma from 6 healthy donors and 9 PDAC patients	[80]
sEVs	4 μL of the filtered serum sample	Locked nucleic acid modified AuNPs@DTNB	miRNA-10b	0.21 fM	In situ detection of target miRNA from exosomes with a detection limit of 0.21 fM	Single marker detection is not suitable for identifying heterogeneous sEVs	Serum samples of 15 healthy people and 15 pancreatic ductal adenocarcinoma patients	[85]
sEVs	0.5 μL of serum sample	Gold nanodots grew on magnetic nanoparticles (MNPs@Au)	sEVs	91.67% sensitivity	The magnetic SERS platform can identify breast cancer patients and healthy people with 91.67% sensitivity and 100% specificity	PCA may not provide detailed molecular information about exosome contents	Serum samples from 6 healthy donors and 14 breast cancer patients	[100]
sEVs	4 μL clinic serum sample	Au@Ag@MBA SERS tags	PD-L1	1 PD-L1^+^ exosome/μL	Personalized exosomal PD-L1 quantification by using a 4 μL clinical serum sample	Single marker detection is not suitable for identifying heterogeneous sEVs	12 healthy donors, 17 NSCLC patients	[84]
sEVs	10 μL of conditioned sEVs	AuNPs coated with Raman reporters and antibodies	Glypican-1, EpCAM and CD44V6	2.3 × 10^6^ particles/mL	Comprehensive evaluation of sEVs heterogeneity with high sensitivity	Method validation with patients’ samples needs to be performed	N/A	[79]
sEVs	N/A	Gold nanostar@Raman reporter@nanoshell	sEVs	27 particles/μL	Sensitive and simple strategy for capture and quantification of sEVs	No further molecular information about exosome contents is detected	Serum samples from 3 healthy donors and 3 liver cancer patients	[101]
Protein	N/A	Au@Raman reporter@Ag nanoparticles	IL-6 and PCT	0.54 pg/mL for IL-6 and 0.042 pg/mL for PCT	Simultaneously rapidly and highly sensitively detect the sepsis biomarkers IL-6 and PCT	Quantifying the concentration of IL-6 and PCT accurately using SERS-based assays might present challenges	55 clinical serum samples of IL-6, and 59 PCT clinical serum samples	[102]
Protein	N/A	Au@Raman reporter@Ag nanoparticles	IL-6	0.453 pg/mL	Detection of IL-6 in human serum samples	Tedious procedure for synthesis of functionalized magnetic nanoparticles	N/A	[103]
Protein	N/A	AgNP-coated silicon wafer	Hypersensitive C-reactive protein	0.01 pg/mL	This label-free method could achieve rapid and sensitive detection of biomolecules	Label-free SERS might face challenges in achieving consistent sensitivity and reproducibility	N/A	[104]

## Microfluidic device-based assay

Since its adoption as an analytical tool in early 2000s, [105] microfluidics has been continuously grown and been widely adopted in a plethora of applications such as single cell analysis, [106] cell and particle isolation, [107] and organs-on-chips [108]. While research on the use of microfluidics continues, the translation of this technology is coming into reality as an in-vitro diagnosis tool. This is because microfluidics enables manipulation of small volumes of samples and reagents, and performs assays including mixing, incubation, and isolation for a successful in-vitro diagnosis assay. Most importantly, it can be easily integrated with diverse biosensing methods to detect disease-related biomarkers.

**Fig. 3:**
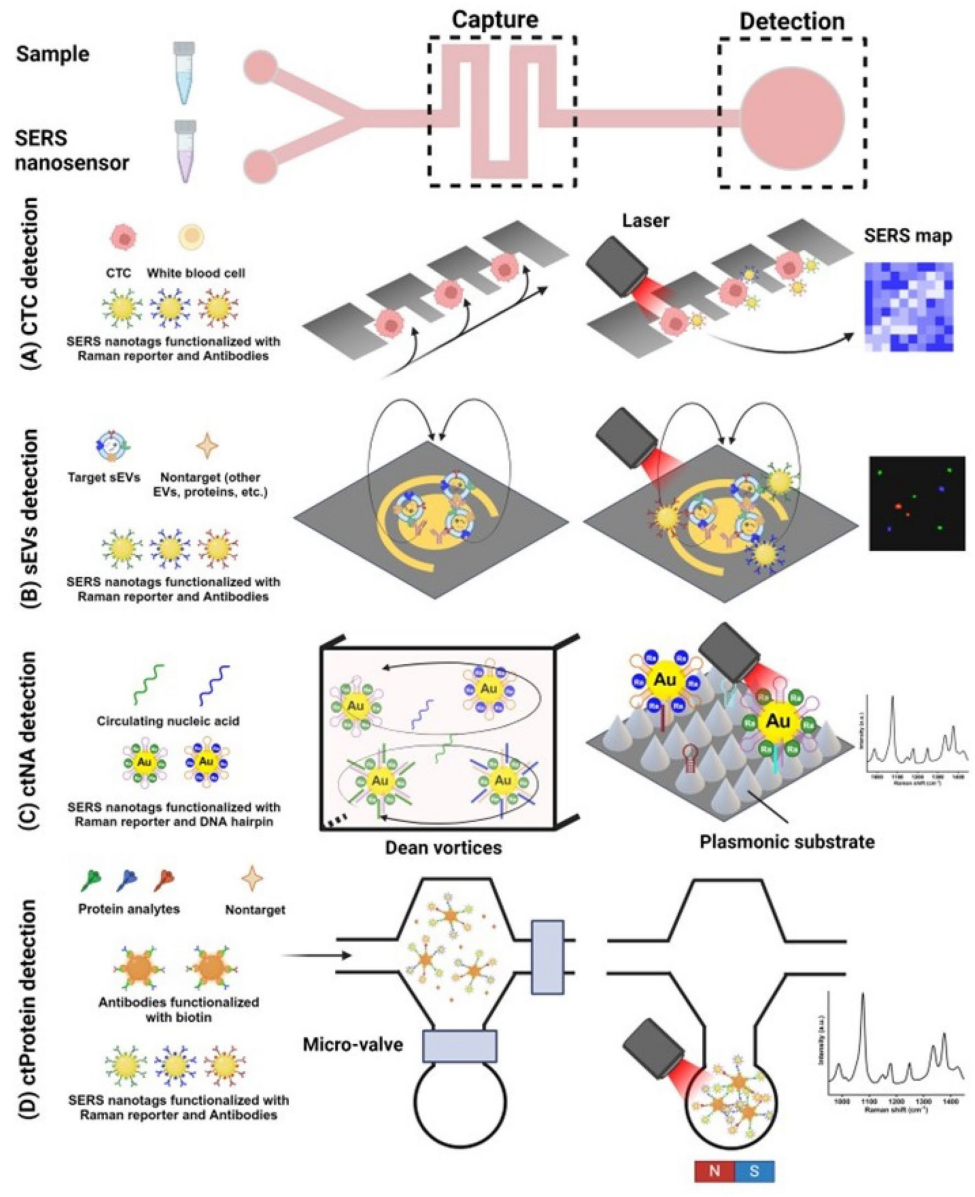
Scheme of microfluidic device-based SERS assays for liquid biopsy detection. A typical microfluidic device should include a capture zone and a detection zone, which can be physically in the same or separate location. **A** CTC detection: an array of traps can capture CTCs based on their differential size followed by SERS mapping on individual captured cells. Reproduced with permission [114]. Copyright 2018, Wiley-VCH. **B** sEVs are much smaller than CTCs and therefore their captures require an efficient mixing at both micro and nano scales; a nanomixing device represents this concept. Reproduced with permission [128]. Copyright 2021, Wiley‐VCH GmbH. **C** Capture of ctNA requires even more efficient mixing, a serpentine channel creating Dean vortices provides such mixing capability which is followed by a further enhanced SERS signal using a plasmonic substrate. Reproduced with permission [131]. Copyright 2023, Dove Medical Press Limited. **D** Protein analytes require precision in capture and detection due to low analyte concentration. This can be achieved by a magnetic capture nanoparticle, flow control through a valving system and detection at small volumes for achieving higher analyte concentration. Reproduced with permission [138]. Copyright 2018, Springer Nature

A microfluidic biosensor can detect trace amounts of circulating biomarkers by *i)* ensuring sensitive and selective capture of biomarkers through frequent ligand-target binding and *ii)* employing a sensitive sensing mechanism to detect captured analytes. SERS can be easily integrated into a microfluidic device in the form of functionalized SERS substrates or SERS nanotags. Such integrated microfluidic SERS will therefore be an attractive candidate for liquid biopsy analysis. The scheme of microfluidic device-based SERS assays for liquid biopsy detection is illustrated in Fig. 3.

### CTCs

CTCs are rare cancer cells that can be as low as 1–10 CTCs per 10 mL of whole blood, [109] isolation of CTCs from blood is therefore essential in CTCs analysis. CTCs are on average larger than other blood cells, and express specific membrane proteins (such as EpCAM). Microfluidic devices leverage these differences to isolate CTCs from blood cells. Captured CTCs will then be used to profile expression of cancer-specific membrane proteins such as EpCAM, cytokeratin (CK), vimentin, and CD133, where the latter two markers used to identify mesenchymal and stem cells phenotypes [110–112]. The multiplexing capability and high sensitivity of microfluidic-integrated SERS biosensors can be used to target multiple membrane proteins at low expression level (such as low EpCAM expressing CTCs) [113].

Integration of SERS biosensors to a microfluidic device depends on the microfluidic isolation mechanism. For example, in a microfluidic trapping device (Fig. 3A), Zhang et al*.* used SERS nanovectors functionalized with detection antibody to target EpCAM, EGFR and HER2 proteins on captured CTCs. These nanovectors harbor unique Raman reporter molecules with distinct Raman peaks allowing independent and multiplexed analysis of the captured CTCs [114]. In another example Gao et al*.* used aptamer functionalized SERS nanotags to perform SERS mapping on individual cells to find epithelial-to-mesenchymal transition (EMT) CTCs. The drawback of this method is that the blood sample is mixed with SERS nanotags on the chip before entering the trapping zone, which increases the chance of channel clogging [115]. An alternative size-based CTC isolation methods is an inertial microfluidic device that isolates CTCs from blood into a separate outlet which is equipped with a SERS substrate [41].

Affinity-based isolation offers an alternative approach with the advantage of avoiding the loss of small CTCs. Cho et al*.* used biotinylated SERS nanotags to capture breast cancer CTCs on streptavidin coated micro-posts for a 5-plex SERS biosensing of MUC1, EpCAM, EGFR, HER2, and CD133 [116]. To increase the chance of CTC capture, Kamil Reza et al*.* used an alternating-current electrohydrodynamic (ac-EHD) device to create nanomixing near the electrode surface for enhanced CTC-capture substrate interaction [117].

An interesting development in SERS biosensor for CTC analysis is a multicolor iron oxide-Au SERS nanotag with dual functions: *i)* selective binding to CTCs expressing a target protein followed by magnetic isolation and *ii)* multiplexed profiling of membrane protein expression through their Raman reporter molecule coating. This method has been proposed by Wilson et al*.* and enabled capture and 4-plexed profiling of EpCAM, HER2, CD44, and IGF1R CTCs from 10X diluted blood samples. [118] The microfluidic device developed in this work uses much smaller sample volume, a high gradient of magnetic field across a narrow channel leading to more effective magnetic isolation, and combines isolation and detection in one device reducing the chances of CTC loss and cross-contamination.

To overcome the limitations imposed by the need to enrich CTCs before SERS detection, a high throughput cell screening using Raman-based flow cytometry can be performed. In this method introduced by Pallaoro et al*.*, cells are focused in a narrow hydrodynamic flow focusing before passing through a laser [119]. This method was further enhanced by Kamil Reza et al*.* that detected three membrane proteins of MCSP, MCAM and LNGFR on melanoma cells via a single flow chamber with sensitivity as low as 10 cells in 1 mL of peripheral blood mononuclear cell (PBMC) [120].

Finally, microfluidic-SERS is a potent tool for functional analysis of single CTCs. An important function of cells is secreting proteins that facilitate cell–cell communication. This is particularly important in analysis of CTCs to find the most invasive phenotypes and to understand the phenotype-function correlation. To quantify this function, single cells can be encapsulated in nano-droplets together with a SERS nanoparticle [121] or a combination of a magnetic capture SERS substrate and a SERS nanotag [122]. The latter strategy allows dual SERS amplification by *i)* turning SERS signal ON through the Raman reporter molecule on magnetic nanoparticles adjacent to AgNPs and *ii)* leveraging the magnetic field-induced spontaneous collection effect, which brings 75 times enhancement for SERS signal [122]. Microfluidic droplet-based SERS biosensors has enabled quantification of critical cancer cells secretum including vascular endothelial growth factor (VEGF) and interleukin-8 (IL-8) that are indicators of tumor growth and expansion [122].

### Circulating tumor-derived sEVs

Like CTCs, sEVs analysis involves isolation and profiling of surface membrane proteins. However, sEVs are significantly smaller than CTCs, measuring at nanoscale range. This intrinsic size difference calls for a new transport strategy that can manipulate these nanovesicles and enhance the contact frequency of EV-capture ligand both at the micro- and nanoscales.

There are several reports on isolation of sEVs based on their size, [123–125] however, on-chip sEVs detection is almost exclusively performed in a microfluidic affinity-based capture configuration (Fig. 3B). One of the most sensitive tumor-derived sEVs detection systems was reported by Wang et al*.* reaching a sensitivity of 1.6 × 10^2^ sEVs/mL. In that work, authors used an efficient mixing strategy where the sEVs sample and the anti-CD63 functionalized magnetic beads were first passed through an array of patterned micropillars. sEVs bound to the anti-CD63 functionalized magnetic beads were then mixed with EpCAM Raman beads and immobilized in a detection zone for SERS detection [126].

A novel microfluidic device “EV phenotype analyzer chip (EPAC)” was introduced by Wang et al*.*, which uses the principles of nanomixing to detect cancer-specific sEVs phenotypes from melanoma patient plasma. AuNPs coated with four Raman reporters and four capture antibodies were used in 4-plex detection of MCSP, MCAM, ErbB3, and LNFGR to monitor their changes during BRAF inhibitor treatment [127]. EPAC-II improved the performance of the previous version by incorporating an antibody cocktail and circulating nanomixing force, which reduces non-specific binding (Fig. 3B). Those improvements led to a 100-fold increase in detection sensitivity (from 10^5^ sEVs/mL to 10^3^ sEVs/mL). One of the challenges in working with this system is operational expertise [128].

In addition, a microfiltration microfluidic device that incorporates a nanoporous polycarbonate track etched (PCTE) membrane sandwiched between microfluidic channels was introduced for osteosarcoma diagnosis, [129] where sEVs and SERS nanotags are pre-mixed and then injected into the tangential flow filtration device for enriching sEVs immunocomplexes and removing free SERS nanotags and plasma biomolecules. This scheme enables 3-plex detection of CD63, EpCAM, and vimentin with sensitivity as low as 2 × 10^3^ sEVs/mL.

### ctNAs

The genetic structure of ctNAs demands microfluidic SERS biosensors that are functionalized with capture oligonucleotides complementary to the ctNA sequence and a signal amplification mechanism to obtain detectable signals (Fig. 3C). Cao et al*.* reported a catalytic hairpin assembly (CHA) strategy where the presence of ctDNA triggers the formation of hairpin duplexes that join the SERS probes and the capture substrate. Capture of SERS probes over the reaction time led to the formation of hotspots that significantly increase the SERS signal [130]. This device contains three functional units: a micromixer for efficient capture of ctNAs on SERS probes, a detection zone, and a capillary network for the passive flow of analytes. The integration of an amplification step either before or after CHA reaction can significantly improve sensitivity, for example, Quian et al*.* and Cao et al*.* used this strategy to detect lung and gastric cancer ctDNAs at attomolar ranges [131, 132]. Interestingly, the sample and SERS nanotags were driven into the microfluidic device chambers through comb-like hydrophilic channels that create capillary flow, thus achieving the passive flow of sample without the need for a pump. This characteristic renders the microfluidic device a suitable choice for point-of-care and low-resource-settings [130].

A recent study in 2024 introduced another pump-free microfluidic SERS biosensor which uses vacuum generated by finger pressure to drive whole blood samples into the device [133]. The blood cells were rapidly removed by passing them through a filter trench before ctDNA capture and detection. Gold nanoprobes functionalized with hairpin structures and Raman reporters were used to detect the target ctDNAs. In the presence of target sequences, the hairpin structures straighten up and become linear in shape, thus increasing the distance between the gold nanorods and the Raman reporters, resulting in the reduction of SERS signal from the Raman reporters. The device successfully detected EGFR E746-A750 mutation in lung cancer patients with sensitivity of 100 fM.

The strategy of altering SERS signals by changing the distance between the Raman reporter molecule and the plasmonic substrate was also employed in the detection of miR-34a, a tumor suppressor molecule [134]. The detection zone in this device was functionalized with miR-34a-specific molecular beacon (MB) which is disrupted in the presence of miR-34a leading to detachment of the Raman reporter from the SERS substrate, resulting in the decrease of SERS signal. An interesting aspect of this device is its recyclability as the SERS substrate can be removed from the device for washing and reusing.

Capturing sEVs miRNAs can also be detected through sEVs capture and lysis on-chip [135]. A multifunctional microfluidic chip first captures sEVs from plasma samples using functionalized magnetic beads, then the captured sEVs are lysed by mixing with a lysis buffer. The lysed sample containing miRNA-21 passes through a detection zone where the miRNA displaces a previously immobilized DNA strand and activates a masked rolling circle amplification (RCA) primer. The subsequent RCA steps produce tandem periodic sequence units, facilitating the capture of numerous SERS nanotags that significantly increase the SERS signal, allowing for detection of miRNA-21 from sEVs with a sensitivity as low as 1 pM [135].

### Circulating tumor-related proteins

The role of microfluidic SERS biosensors in detection of circulating proteins are *i)* ultra-sensitive detection in a wide protein concentration range of ng/mL to fg/mL, and *ii)* meeting the highly multiplexed detection requirement of circulating protein profiling, which is essential in obtaining a high-resolution picture of the tumour status.

Microfluidic channels can create a spatial pattern to achieve individual zones for parallel detection. In a study by Zheng et al*.*, a microfluidic stamping device was first used to create patterns of AgNPs functionalized with capture antibodies against breast cancer specific biomarkers CA153, CA125 and CEA [136]. Subsequent stamps were made to introduce the sample followed by SERS nanoprobes for the detection. This strategy was also used for 3-plex detection of IL-6, IL-8 and IL-18 cytokines, which are stimulants of tumour cell proliferation, malignant transformation, and progression [137].

Several strategies have been employed to increase the capture of circulating protein and improve assay sensitivity. First, is a microfluidic SERS biosensor that comprised of a mixing unit to selectively capture proteins on antibody-conjugated magnetic nanochains (Magchain) followed by opening a microfluidic valve for mixing with SERS-encoded probes to form sandwich immune complexes (Fig. 3D) [138]. The mixed sample is then exposed to a magnetic field which routes Magchains with attached proteins to a Raman detection zone. This device allowed detection of prostate-specific antigen (PSA), carcinoembryonic antigen (CEA), and α-fetoprotein (AFP) circulating proteins with high sensitivity of 10 pg/mL. The second strategy is ac-EHD device developed by Kamil et al., which benefits from the increased surface area offered by graphene oxide substrate and micromixing through electrohydrodynamic flow for 4-plex detection of HER2, EGFR, MUC1, MUC16 at the high sensitivity of 10 fg/mL [139].

Finally, microdroplet systems also offer increased mixing efficiency for improved capture and sensitivity. In this strategy, the sample, capture magnetic beads and SERS nanotags are first mixed into droplets that follow a winding channel that creates a chaotic advection for mixing the reagents. A Y-shaped splitting junction breaks up the droplet to separate the immunocomplexes from the residual SERS nanotags. The immunocomplexes are then collected in a separate chamber and analysed via SERS.

In summary, microfluidics-SERS provides benefits of low sample consumption, portability for point-of-care testing, delicate manipulation of input analytes, and superior integration with SERS biosensors. Efficient capture and detection are at the core of all microfluidic-SERS biosensors. However, each circulating biomarker has its own requirement, as presented in Table 3. First, the design of the microfluidic device should be tuned to enable manipulation of input sample. For instance, CTCs need to be isolated from blood samples, and EVs and ctNAs need to frequently collide with the functionalized capture substrates through one of the mixing strategies. Second, the microfluidic device should deliver SERS nanosensors to the captured analytes uniformly while simultaneously preventing their clogging and aggregation. These two criteria directly affect the device’s sensing capability quantified via assay sensitivity, specificity and multiplexity. Ligand conjugated SERS nanoparticles need to efficiently find their target (i.e. high sensitivity), while removing the chance of non-specific binding through washing steps (i.e. high specificity). As a final evaluation criterion, it is important to have a holistic view of each microfluidic method and its advantages in clinical application. Assay time, ease of manipulation, SERS interface integration, minimal contamination, and flexible and automated operation are all important factors in assessing a microfluidic SERS biosensor platform for a specific application.

**Table 3:** Microfluidic SERS biosensors for detection of circulating markers for cancer diagnosis

Target	Device type	Sensitivity (LOD)	Specificity	Multiplexity	Sample	SERS sensor	Merits	Demerits	Clinical application	Ref.
CTCs	Trap	2 spiked CTCs/100 µL of 10 × diluted blood	EpCAM, EGFR, HER2	3-plex	10X diluted blood	Au@Ag core/shell nanovectors	Size-based capture of heterogeneous CTC subpopulations	Diluted sample and low flow rate	Identifying breast cancer subtypes	[114]]
CTCs	Trap	14 CTCs/1 mL whole blood	EpCAM, Vimentin	2-plex	Whole blood	AuNPs	Direct use of whole blood, high capture purity and efficiency	Chance of clogging	Hepatocellular carcinoma (HCC)	[115]
CTCs	Trap	NA	EpCAM, EGFR, HER2	3-plex	Culture medium	Au@Ag core/shell	Simultaneous biochemical and phenotypic profiling	Removal of excess SERS nanoprobes	Breast cancer	[140]
CTCs	Immunomagnetic	1000 CTC spiked in 10 × diluted blood	EpCAM, HER2, CD44, IGF1R	4-plex	10 × diluted blood	Magnetic multicolor iron oxide-gold core–shell nanotags	Immunomagnetic enrichment with high specificity, choice of clinically relevant biomarkers	No study in sensitivity and specificity for CTC isolation	Breast cancer	[118]
CTCs	Microfilter	2 cells/mL	Folate receptor	Single plex	Diluted blood ratio of 1:2	Crystal amorphous core–shell B-TiO_2_ nanoparticles	Quick assay time of 1.5 h	Sample clogging, challenge in scanning for CTCs	Cancer progression and patient prognosis	[141]
CTCs	Continuous flow	10 cells spiked in 1 mL PBMC sample	Melanoma markers MCSP, MCAM, and LNGFR	3-plex	PBMC isolation	SERS nanotags	Combined surface and intracellular protein profiling	CTCs loss during PBMC isolation	Dynamic monitoring of CTC following treatment	[120]
CTCs	Continuous flow	N/A	EpCAM	Single plex	Mix of MCF7 cells & WBCs	Multicore gold@Ag@SiO_2_ nanoaggregates	Rapid and reproducible, integration for Raman cell sorting	Low throughout, no sensitivity report	Large-scale profiling of cell in blood	[142]
CTCs	Continuous flow	1 cancer cell in 100 non-cancerous cells	Neurophilin-1	2-plex	Prostate cancer cells	Ag@thionin nanoparticle dimer	Highly reproducibility	Low throughput, low reported sensitivity	Large-scale profiling of cell in blood	[119]
CTCs	Immunocapture and trap	100 breast cancer cells	HER2, CD133, EGFR, EpCAM, MUC1	5-plex	Spiked cells in WBC from 4 mL blood	Raman active nanoprobes	Combined isolation, detection and release, high multiplexing	N/A	Isolating CD133 + cancer stem cells	[116]
CTCs	Immunocapture,	100 SKBR3 cells	HER2	Single plex	SKBR3 cells suspended in PBS	SERS nanotags functionalized with MBA	Integration with graphene oxide and AC-EHD	No information on capture efficiency	CTC heterogeneity profiling	[117]
CTCs	Magnetic immunocapture	1–5 CTCs/mL of blood plasma	EpCAM	Single plex	Prostate cancer cells spiked in plasma	Ag@Fe_2_O_3_/p-MBA and SERS active substrate	Dual nanoparticle and SERS-active substrate Raman signal enhancement	Signal from multiple cells not single cell	Profiling CTCs of wide EpCAM expression levels	[113]
CTCs	Droplet microfluidics	10^5^ cells/100 µL solution	EpCAM, Vimentin, CD45	3-plex	SKBR3 mix with gold nanostar	Gold nanostar @1-NAT@SiO_2_	Suitable for high throughput screening	Sophisticated system operation	High throughput screening	[143]
CTCs	Droplet microfluidics	1.85 × 10^6^ cells/mL	Vascular endothelial growth factor (VEGF)	Single plex	Cells, stimulant, and nanoparticles mixed in a droplet	Capture via SiO_2_@biotinylated Ab-VEGF mAb Detection via AgNP@4-MBN-VEGF-pAb	Hotspots formation through aggregation, reduced non-specific binding, real-time monitoring	Encapsulated cells should be collected off-chip for further analysis	High through put profiling and sorting	[144]
CTCs	Droplet microfluidics	10^6^ cells/mL	Glycan N-acetyl neuraminic (sialic) acid	Single plex	PC3 mix with SERS nanoprobe	Lectin wheat germ agglutinin (WGA) gold nanoparticle	Fast coarse mapping and slow detailed interrogation, addressable droplets	Sophisticated system operation, lack of washing step	Cell membrane carbohydrate as oncology targets	[121]
CTCs	Droplet microfluidics	10^6^ cells/mL	VEGF and Interleukin-8 (IL-8)	2-plex	Stimulated cells mix with Ag and MN NPs	AgNPs@Ab1 for capture and MNs@reporter-Ab2 for detection	Magnetic field- spontaneous collection effect- 75 times SERS enhancement	Sophisticated system operation, no washing step	Indicators of tumour growth and expansion	[122]
EVs	Immunomagnetic	1.6 × 10^2^ particles/mL	EpCAM	Single plex	20µL filtered serum of prostate patients	CD63 magnetic beads for EV capture and EpCAM-functionalized Raman beads	High capture efficiency and sensitivity	Magnetic bead aggregation, clogging, false signal	Sensitive PCa diagnosis	[126]
EVs	Immunocapture and EHD	10^5^ EVs/mL	MCSP, MCAM, ErbB3, LNFGR	4-plex	SK-MEL-28 EVs spiked in healthy plasma	AuNPs	EHD flow causing nanomixing for efficient EV capture	Sophisticated operation	Detecting melanoma -specific EV phenotype	[127]
EVs	Immunocapture and EHD	10^3^ EVs/mL	MCSP, MCAM, CD61, CD63	4-plex	Serum EV, 20 early-stage melanoma patients	SERS nanotags: AuNPs functionalized with MBA, TFMBA, DTNB, and MPY	EHD flow causing circulatory fluid flow nanomixing for efficient EV capture	Sophisticated operation	Detection of early-stage melanoma	[128]
EVs	Membrane filtration	2 × 10^3^ EVs/mL	CD63, EpCAM, Vimentin	3-plex	Plasma	AuNPs@MPY or MBA or TFMBA	Simple operation	Potential nanotag aggregation and clogging	Osteosarcoma diagnosis	[129]
EVs	Immunocapture	2 × 10^6^ EVs/mL	EpCAM, HER2	Single plex but used for multiple markers	Plasma	Anti-CD63 antibody for exosome capture, gold nanorods coated with QSY21 Raman reporters	Simple pipette-based assay	Low sensitivity	HER2 + Breast cancer diagnosis	[145]
ctDNA	µmixer; CHA^a^ binding SERS probe to substrate	2.26 aM and 2.34 aM	NSCLC lung cancer ctDNA (TP53 and PIK3CA-Q546K)	2-plex	Serum	Au-AgNSs@4-MBA@HP1-1 and Au-AgNSs@DTNB@HP^*^1–2; Ag nanoshells as capture substrate	Fast reaction time; Pumpless device using capillary force, radial design for parallelization	Lack of microfluidic device characterization	Early in NSCLC lung cancer screening	[130]
ctDNA	µmixer & magnetic enrichment, CHA^a^ bind SERS probe to mag. bead	3.116 aM and 3.921 aM for BRAF & KRAS	BRAF V600E and KRAS G12V, NSCLC^a^ ctDNAs	2-plex	Serum	Pd-Au core–shell nanorods Pd-AuNRs@DTNB@HP1-1 and Pd-AuNRs@4-MBA@HP1-2	Fast reaction time Pumpless device	Dependency on collection capability of magnetic beads	Clinical diagnosis of lung cancer NSCLC	[146]
ctDNA	µmixer EASA^a^ and CHA^a^ signal amplification	2.16, 2.33, 2.47, 3.15 aM for BRAF PIK, KRAS and TP53	BRAF V600E, PIK3CA Q546K, TP53, and KRAS G12V	2-plex	Serum	Au-AgNBs@HP, Au-TPP, DTNB, HP-DNA-functionalized Au nanocone arrays as capture substrates	Pumpless device	Dual amplification adding to assay complexity and time	Diagnosis treatment monitoring of lung cancer patients	[131]
ctDNA	Micromixer; CHA^a^, HCR^a^ as signal cascade amplifiers	PIK3CA E542K (1.26 aM) and TP53 (2.04 aM)	PIK3CA E542K and TP53 (two GC-related ctDNAs)	2-plex	Serum	Cu_2_O@DTNB@HP, Cu_2_O@4-ATP@HP, 4-MBA-labelled AuNB array as capture surface	Portability, simultaneous analysis of six sample	Complex chemistry	Early diagnosis of Gastric cancer	[132]
ctDNA	Blood filter trench, DNA hairpin functionalized surface	100 fM	EGFR E746-A750 mutation	Single plex	Whole blood	Au@Ag nanorods	Using whole blood directly in the chip, amplification-free	Microfluidic isolation of blood cells not clear	EGFR mutation detection in lung cancer patients	[133]
Exosome miRNA	Magnetic enrichment and µmixer; RCA^a^ & TSA^a^	1 pM	MicroRNA-21	Single plex	EVs derived from MCF7 cultures	SERS probe: AuNP@MBA-tyramine; Gold nanoclusters as plasmonic substrate	Multi-functional device for capture, lysis and detection	No use of clinical sample	Diagnosis of breast cancer	[135]
Single cell miRNA	Nano-droplet encapsulation,	10 pM	MicroRNA-21	Single plex	Cancer cells HeLa, HepG2 and MCF-7	AuNP-ssDNA ROX probe- Capture ssDNA	Lysis-free, eliminating time-consuming for RNA isolation	Lack of multiplexity	Capture of genetic heterogeneity in cancer cells	[147]
miRNA	Functionalized SERS array assembled microfluidic device	1 pM	MicroRNA-21	Single plex	Serum	Porous anodic aluminium oxide (AAO) SERS substrate, Au-MBA@Ag core–shell nanoparticles; ssDNA in between for miRNA capture	Simple device design, low sample requirement; false positive or false negative can be avoided by using dual SERS mode	Lack of multiplexity	Cancer diagnosis from serum	[148]
miRNA	Functionalized SERS substrate molecular beacons,	5 fM	MicroRNA-34a	Single plex	Addition of miRNA in serum solution	miR-34a-specific molecular beacon (MB) for capture, Cy3 as a Raman reporter	Uniform signal, reproducibility and high sensitivity, recyclability	Requirement of washing the device for further use	Tumour suppression biomarker detection	[134]
Protein	Alternating-current electrohydrodynamic (ac-EHD)	100 fg/mL	HER 2 and Mucin 16	2-plex	Proteins spiked in serum	AuNPs@DTNB@anti-HER2, AuNPs@MMC@anti-Mucin 16	Superior sensitivity due to nano-flow; graphene oxide functionalization increasing the active surface area	Complex operation, nonuniform capture and detection due to flow; chance of clogging	Tumour protein biomarker detection	[117]
Protein	Spatial multiplexing functionalized SERS substrates	CA153, CA125, 0.01 U/mL & CEA 1 pg/mL in serum	CA153, CA125, and CEA	3-plex	Spiked in 5% serum solution	Ag NPs@DTNB, 4MBA and 2-naphthalenthiol (2NAT)@Ab	Spatial multiplexing allowing easily distinguishing between captured proteins	Complicated design	Breast cancer specific biomarker detection	[136]
Protein	Microdroplets	0.1 ng/mL for both	Free prostate-specific antigen (f-PSA) and total PSA (t-PSA)	2-plex	Serum samples from prostate cancer patients	Capture antibody-conjugated magnetic bead, antibody-conjugated SERS nanotag	Clogg-free operation, rapid and efficient mixing enabled by segmented droplet flow	Complex design and operation	Accurate diagnosis of prostate cancer	[149]
Protein	Embedded Ag-Au bimetallic Antibody functionalized substrates	3.8, 7.5, and 5.2 pg·ml − 1 for IL-6, IL-8, and IL-18	IL-6, IL-8, and IL-18	3-plex	Plasma	AuNP@Raman reporter@detection antibody	Flexibility between parallel vs simultaneous detection schemes	Potential sample handling errors	Markers proliferation, malignant transformation, and progression	[137]
Protein	Alternating-current electrohydrodynamic (ac-EHD)	10 fg/mL	HER2, EGFR, MUC1, MUC16	4-plex	Proteins spiked in serum	Au SERS nanotags functionalized with MBA, DTNB, MMC, TFMBA	Superior sensitivity due to nano-flow	Requirement for flow rate adjustment for efficient capture	Detection of breast, lung, and ovarian cancer biomarkers	[139]
Protein	Magnetic isolation	10 pg/mL	PSA, CEA, AFP	3-plex	Proteins spiked in serum	Ab1 conjugated magnetic nanochains (Magchains), Au@Ab2 nanorods	On-chip mixing, incubation, and detection	Automated operation requires microvalve integration	Diagnosis of prostate, colorectal and liver cancer	[138]

^a^*CHA* catalytic hairpin assembly, *NSCLC* Non-small cell lung cancer, *EASA* enzyme-assisted signal amplification, *HCR*, hybridization chain reaction, *HP* Hair pin, *RCA* Rolling circle amplification, *TSA* tyramine signal amplification

## Paper-based SERS assay

The use of paper as a substrate for detection assays has a long history that extends over many centuries, with some of the earliest instances being traced as far back as the seventeenth century [150, 151]. Over time, numerous scientific advancements have paved the way towards the development of modern paper-based detection assays for increasingly complex applications. The invention of paper chromatography in 1944 laid the groundwork for the paper chromato-electrophoresis radioimmunoassay in 1959, which introduced the immunoassay technology that led to the invention of the pregnancy test: the first at-home paper-based point-of-care (POC) test to reach the market in 1985 [151–155].

Since then, paper-based detection assays have continued to evolve and diversify into established FDA-approved tools for diagnosis and disease monitoring. Paper substrates are often the preferred platform for designing POC tests due to their cost-effectiveness, biocompatibility, adaptability, and portability, which are attributes that satisfy most of the ASSURED criteria (Affordable, Sensitive, Specific, User-friendly, Rapid and robust, Equipment-free, and Deliverable to end users) outlined by the World Health Organization (WHO) in 2006 to evaluate the effectiveness of POC devices [156–158].

**Fig. 4:**
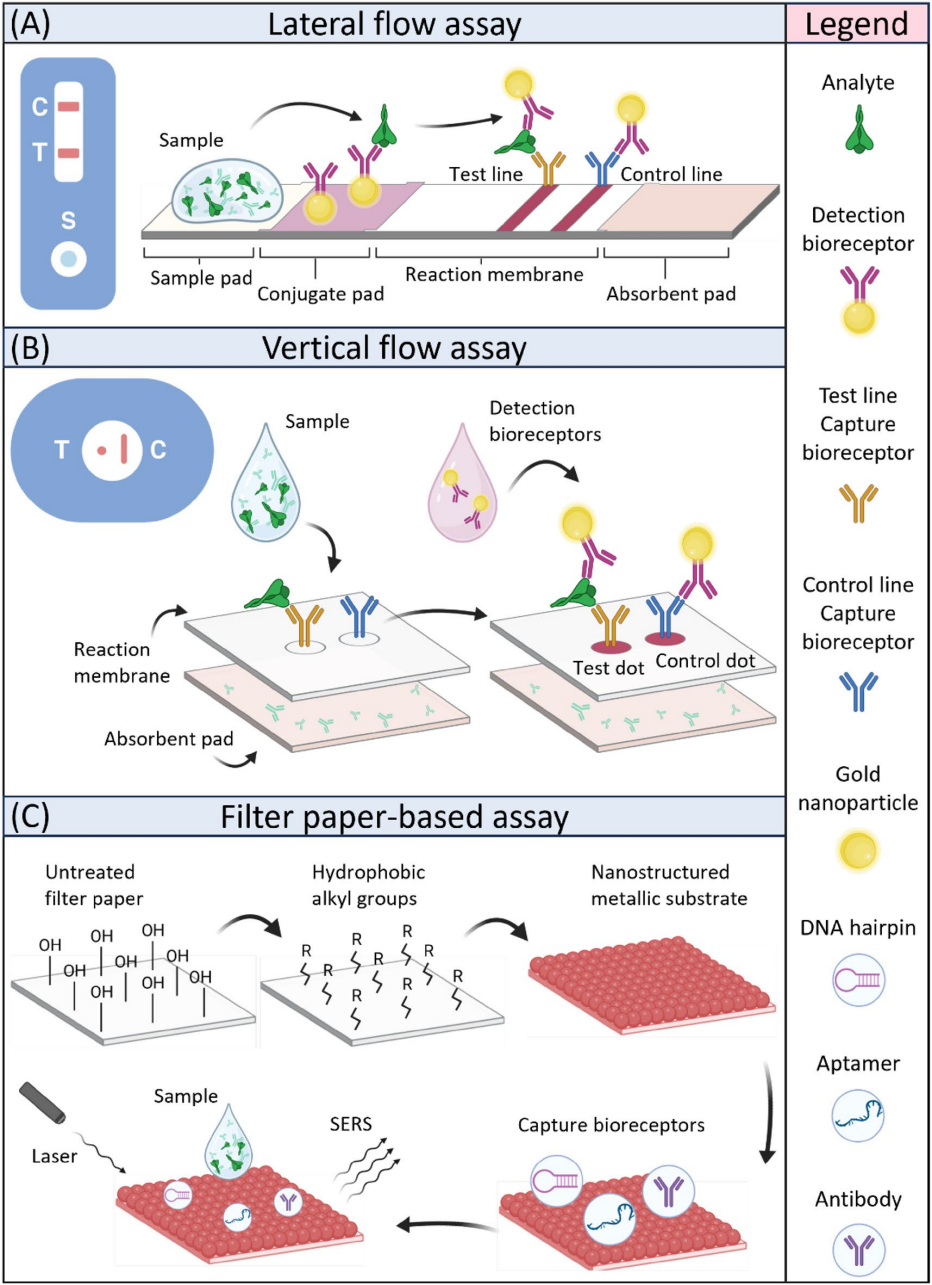
Scheme of paper-based SERS assays for liquid biopsy detection representing **A** a lateral flow assay device (left) and a lateral flow assay strip (right) depicting the lateral movement of the sample from the sample pad to the conjugate pad, nitrocellulose membrane, and absorbent pad; **B** a vertical flow assay device (left) and a vertical flow assay structure with the reaction membrane/substrate and absorbent pad arranged vertically (right), depicting the addition of the sample to the device followed by detection using gold nanoparticle bioreceptors; and **C** the modification of filter paper with alkyl groups that allows the attachment of gold nanoparticles (red) on the substrate to enhance Raman signals

These devices come in various forms (Fig. 4), including as lateral flow assays (LFAs), vertical flow assays (VFAs), microfluidic paper-based analytical devices (µPADs), and simple filter paper-based assays. LFAs are the most common type of paper-based detection assays commercially available, this is the format of most pregnancy and COVID-19 rapid antigen tests (RATs). A typical LFA (Fig. 4A) is composed of a sample pad, a conjugate pad, a nitrocellulose (NC) reaction membrane, and an absorbent pad, all fixed onto a strong backing card for support and durability. Liquid sample is first added to the sample pad, and as the name suggests, it travels laterally along the assay due to the capillary action of the porous substrates, subsequently, the sample enters the conjugate pad where it encounters detection bioreceptors that bind to the sample and create bioreceptor-sample complexes. Gold nanoparticles (AuNPs) are the most common detection bioreceptors used in LFAs, they are conjugated with antibodies against the target analyte and dried in the conjugate pad. Upon contact with the liquid sample, the AuNPs are re-solubilized and released from the pad to bind to the target analyte via the antibodies, the bioreceptor-sample complexes then continue to travel along the assay into the NC membrane where the test and control lines are located. The test line contains capture bioreceptors that bind to the target analyte. When the bioreceptor-sample complex reaches the test line it becomes immobilized on the NC membrane, thus forming a sandwich-like structure linking the AuNPs, the detection antibody, the target analyte, the capture antibody, and the NC membrane together. The accumulation of various AuNPs onto the test line produces a red signal that indicates the presence of the target analyte. If no signal is produced, then there was no target analyte to form linkages between AuNPs and the NC membrane. Control lines are added as quality control, they are designed to produce a signal regardless of the presence of the sample.

VFAs help mitigate the “hook effect” that can cause false negatives in LFAs when used for samples with very high concentrations of analyte [159, 160]. The pads and membranes of VFAs are organized vertically so that the sample traverses across the conjugate pad, the NC membrane, and the absorbent pad (Fig. 4B). Some VFAs consist of only a NC membrane and an absorbent pad, here the AuNPs detection bioreceptors are pre-mixed with the sample beforehand and then added to the device. On other occasions, the untreated sample is added first, then a conjugate pad is placed over the VFA and the AuNPs are released with buffer.

µPADs were developed more recently in 2007 and quickly became one of the most researched paper-based platforms due to their affordability and portability [151, 161]. Their principle focuses on patterning paper to create microchannels that direct liquid reagents and samples through the device. This was a groundbreaking technique due to the increased control that allowed for the development of increasingly complex devices. In contrast, filter paper-based assays (Fig. 4C) are usually simple devices composed of functionalized filter paper with at least one test zone.

The first detection assay to combine paper substrates and SERS was introduced in 2010 by Yu and White, and a few years later in 2014, Li et al*.* pioneered the development of the first SERS-based immunochromatographic assay that was pivotal for the development of various forms SERS-based LFAs commonly studied today [162, 163]. The integration of SERS in paper-based assays remarkably improved sensitivity and allowed for the quantification of analytes, which is not possible with the colorimetric formats commonly used. Consequently, assay portability and user-friendliness were negatively affected by the need for external equipment to read and measure Raman signals. Although this disadvantage could soon be mitigated with the introduction of portable SERS readers, the equipment requirements still make it difficult for SERS-based assays to meet ASSURED criteria for POC tests [156–158, 164].

Nevertheless, increased assay sensitivity is particularly important for the detection of scarce biomarkers, like in the early detection of diseases where biomarkers might not be as abundant. Cancer is a very common disease in which early diagnosis is paramount for a good prognosis. As such, highly sensitive techniques would be very helpful for disease monitoring and the reduction of mortality rates. This section describes recent advancements in paper-based assays that use SERS technology for the detection of common liquid biopsy biomarkers, including CTCs, ctNAs, sEVs, and circulating cancer protein biomarkers. The scheme of the paper-based SERS assay for liquid biopsy detection is illustrated in Fig. 4.

### CTCs

Although CTCs have been the focus of various paper-based devices, the integration of SERS with paper-based devices for the detection of CTCs remains unexplored to date. Moreover, among the limited number of paper-based devices for CTCs detection, the majority were validated with human serum samples spiked with cancer cell lines. Therefore, these devices might not be as effective when used with clinical patient samples due to the complexity of blood samples [165–167]. This might be due to the markedly low abundance of CTCs in blood, with a concentration of 1–10 to 10–100 CTCs per mL of whole blood, [109, 168, 169] and the evident difficulties in procuring real CTCs patient samples.

### ctNAs

Colorimetric LFAs perform best with uniform nanoparticles with smooth spherical surfaces, while nanoparticles with rough surfaces are best suited for SERS-based LFAs [170]. Li et al*.* recently developed two LFAs for the detection of ctDNA and miRNA in clinical serum samples [171, 172]. The SERS substrates for both LFAs were palladium gold core–shell nanorods (Pd-AuNRs) conjugated with Raman reporters, and detection was coupled with catalytic hairpin assembly (CHA) for signal amplification. Tips, edges, and rough surfaces in nanoparticles serve as “hot spots” for generating strong SERS signals. Therefore, rougher nanoparticles such as gold nanostars (AuSts) and Pb-AuNRs lead to higher enhancement factors (EF) of the Raman signal compared to gold nanospheres [171–175]. The Raman signal from Pb-AuNRs is further enhanced with CHA, an amplification-free method that increases the binding of Pb-AuNRs to the test line. Pb-AuNRs are coupled with biotinylated hairpin structures that unravel after hybridization with sample strands to expose the biotin molecule, which binds to streptavidin immobilized on the NC membrane, thus driving the accumulation of Pb-AuNRs on the test line. The sample strands are freed by the introduction of another sequence with higher affinity to the hairpin structures, in this way, the hairpin structures remain open with the exposed biotin molecule, while the sample strands are recycled through the system. The accumulation of Pb-AuNRs on the test line is therefore independent from the number of sample strands present, thus greatly amplifying the generated signal.

These principles were applied for the detection of TP53 and E545K ctDNA biomarkers, and miR-106b and miR-196b biomarkers in serum samples from laryngeal squamous cell carcinoma patients [171, 172]. The LOD for both LFAs reached attomolar levels ranging from 23.1 to 61.3 aM within 30–45 min, with results comparable to qRT-PCR [171, 172]. Mao et al*.* applied the same CHA principle in another LFA for the multiplexed detection of miR-21 and miR-196a-5p on a single test line, achieved by using two SERS probes that can be distinguished in the same Raman spectrum due to their distinct Raman peaks. Although this assay demonstrated higher LODs ranging from 2.2 ‒ 3.3 pM, it offered the distinct advantage of the simultaneous detection of two biomarkers in non-invasively collected urine samples [176].

Multiple hairpin sequences and long preparation times for SERS probes might negatively affect the cost-effectiveness and accessibility of these LFAs. Although sensible, the stability of the SERS probes and hairpin structures within LFA materials remains to be elucidated. Moreover, the preparation processes of clinical specimens were not detailed in the studies, so it is unclear whether any type of nucleic acid isolate or enrichment was performed prior to adding samples to the LFAs.

### Circulating tumor-derived sEVs

Recently, Su et al*.* developed two types of paper-based devices for the quantification of breast cancer exosomes in serum: a 3-plex VFA and a 2-plex LFA [177, 178]. These two platforms are vastly different, with distinct advantages and disadvantages. The VFA was made to quantitatively profile serological exosomes from different breast cancer subtypes through the detection of exosomal surface proteins carcinoembryonic antigen (CEA), human epidermal growth factor receptor 2 (HER2), and Mucin 1 (MUC1) [177]. AuSts coupled with the Raman reporter *p*-nitrothiophenol (*p*-NTP), and anti-CD63 aptamers were used as SERS probes, while three different test zones were coated with capture aptamers against HER2, MUC1, and CEA [177]. The different Raman signals from the test zones were used to measure the proportions of exosomal surface proteins and discriminate between exosomes from different origins. This approach was applied to analyze samples containing a mixture of exosomes derived from four breast cancer cell lines (MCF-7, SKBR-3, MDA-MB-231 and BT474) in a 50% FBS medium, as well as four breast cancer subtypes (luminal A/B, HER2, triple negative) in human serum samples [177]. Although a good discrimination of exosomal protein expression profiles was observed, it is important to note that these findings were obtained using samples with known exosomal origins. This becomes more evident through the examination of the profiles of HER2 and luminal B serum samples, which were nearly indistinguishable from one another. A blind study with sample mixtures of unknown proportions or breast cancer subtypes would be beneficial to accurately measure the efficacy of this assay in profiling exosomes. Another issue posed by this approach, also observed in other multiplexed assays, is that a sample might contain a heterogenous mixture of exosomes from different origins or cancer subtypes and that each type of exosome can contribute to the final Raman signal differently. The strongest Raman signals from the most prominent group of exosomes could overshadow the more subtle signals from the minority groups, thus certain exosomes in the mixture might be overlooked in the analysis.

Su et al*.*’s LFA sought to overcome this issue by integrating two spectrally different SERS probes made of AuSts coupled with distinct Raman reporters and aptamers against HER2 and MUC1 on the conjugate pad, while coating the test line with anti-CD63 aptamers [178]. Similar to Mao et al*.*, HER2 and MUC1 were simultaneously detected on a single test line using these two SERS probes with distinct Raman reporters [176, 178]. Su et al*.* took this a step further by using the combined Raman signals of the SERS probes to generate distinct Raman signatures that can differentiate between exosomes derived from SKBR-3 and MCF-7 cell lines (SKBR and MCF exosomes) [178]. A mixture of SKBR and MCF exosomes can result in a total of four Raman signals on the test line: SKBR-3 exosomes + HER2 /MUC1 probes, and MCF exosomes + HER3/MUC1 probes. Since there are only two Raman reporters here, there is no way to distinguish between the Raman signals from SKBR and MCF exosomes. Multivariate spectral unmixing analysis solved this issue by separating the combined Raman spectra of the two SERS probes into two separate individual spectra that each represent a different type of exosome. Traditional methods, similar to Su et al*.*’s VFA, identify the proportions of multiple biomarkers in the sample as whole without considering that the sample can contain different types of exosomes that each contribute to the final Raman spectra differently. In contrast, this approach can differentiate between exosome subtypes in a complex matrix, thus providing a more comprehensive analysis of the sample [178]. Low LODs of 3.27 × 10^6^ SKBR exosomes/mL and 4.80 × 10^6^ MCF exosomes/mL were observed, although this was measured in 50% FBS instead of human serum. While the assay required a large volume of 100 µL isolated exosomes, it is noteworthy that the procedure was completed in only 15 min and validation with 39 breast cancer human serum samples yielded results in line with ELISA [178].

### Circulating tumor-related proteins

Various studies have reported the potential improvement in diagnostic efficacy of detection assays capable of identifying multiple protein biomarkers, as opposed to assays focused on a single protein biomarker [179–182]. Multiplexed paper-based SERS assays are often designed by immobilizing distinct capture bioreceptors to multiple test zones, each aimed at detecting specific biomarkers. In contrast, alternative designs combine different capture bioreceptors into a singular test zone for the simultaneous detection of multiple biomarkers. In 2019, Chen et al*.* reported the first multiplexed SERS-based VFA by immobilizing capture antibodies against CEA, AFP, and PSA on a single test dot [183]. The assay procedure was completed in 7 min with as little as 10 µL of serum, and, although only 5 replicates were tested, consistent results between the VFA and ELISA were observed with prostate cancer human serum samples. Low LODs of 0.260–0.370 pg/mL were obtained using Raman dye (RD) encoded core–shell (Au@Ag) SERS nanotags that further enhance Raman scattering through the nanogaps between the gold core and the silver shell [183]. This creates the plasmon coupling effect that amplifies the electric field on the nanoparticle surfaces and leads to significantly enhanced Raman signals. However, since the LOD was determined without the use of biological samples, it is probable that clinical serum specimens will result in higher LODs [183]. In 2020, Xia et al*.* also developed a multiplexed single-test line LFA for the detection of squamous cell carcinoma antigen (SCCA) and cancer antigen 125 (CA125) in cervical cancer human serum samples [184]. This reduces variability in LFAs caused by the different distances between test lines and the sample pads [185]. The assay was completed within 20 min with a LOD of 7.37 pg/mL for SCCA and 8.10 pg/mL for CA125 [184]. While Xia et al*.*’s assays did not display the same level of sensitivity as those developed by Chen et al*.*’s, it is noteworthy that Xia et al*.*’s LOD was measured and validated against ELISA with 120 serum samples [183, 184].

Lu et al*.* proposed a filter paper-based SERS assay with a single test zone coated with capture antibodies against SCCA and osteopontin (OPN) for the detection of cervical cancer human sera samples [186]. Notably, an interesting mechanism for Raman enhancement was introduced by coating the filter paper with gold nanoflowers (AuNFs) coupled with the capture antibodies on the test zone [186]. Since there is no conjugate pad, the sample was added directly to the filter paper followed by the addition of SERS immunoprobes after an incubation period of 1 h [186]. Gold-silver nanoshuttles (Au-AgNSs) in the SERS immunoprobes are elongated with arrow-like structures at both ends of the particles, which serve as hot spots for enhancing the electric field and increasing the Raman signals [186]. This signal is further enhanced by the plasmonic coupling effect from the close distance between the AuNFs on the filter paper and the Au-AgNSs in the SERS immunoprobes. Although this assay seems simple, it has a lengthy operation time of > 2 h and a combined fabrication time of > 18 h [186]. Noteworthy results were nevertheless obtained, with low LODs recorded at 8.63 pg/mL for SCCA and 4.39 pg/mL for OPN [186]. Furthermore, a strong correlation between ELISA and this approach was established during validation with cervical cancer serum samples [186].

Fan et al*.* and Peng et al*.* took this approach one step further by integrating conventional SERS-LFAs with a plasmonic internal standard (PIS) made of plasmonic nanoparticles embedded into NC membranes [187, 188]. In this new LFA, called PIS-LFA, sample molecules travel to the conjugate pad to bind with the SERS immunoprobes and form complexes, which then travel along the NC membrane to encounter the PIS nanoparticles as well as the test and control lines. The nanogaps between the PIS nanoparticles on the NC membrane and the SERS immunoprobes create the plasmon coupling effect that significantly enhances Raman signals [187, 188]. The plasmonic nanoparticles on the PIS also generate a Raman signal independently from the SERS immunoprobes, this serves as a constant background signal that can be used as a normalization factor by calculating the Raman peak intensity ratio between the SERS immunoprobes and the PIS [187, 188]. This reduces signal variation from external factors, thus improving overall assay sensitivity and reproducibility. An advantage of Fan et al*.*’s design was the addition of capture antibodies against CEA and neuron-specific enolase (NSE) onto a singular test line, thus allowing the simultaneous detection of both biomarkers [188]. Consistent results in the detection of CEA and NSE with early-stage lung cancer human serum samples were observed using both the PIS-LFA and electrochemiluminescence immunoassays (ECLIA) [188]. Although low LODs of 39.0 pg/mL and 46.0 pg/mL were achieved for both CEA and NSE, respectively, it is important to note that these measurements were obtained without the use of biological samples [188].

Nanostructures of various shapes and sizes have been engineered to increase the number of nanogaps or hot spots, with the goal of significantly enhancing Raman scattering. This was observed when She et al*.* used the curiously shaped raspberry-like Fe_3_O_4_@Au magnetic nanoparticles (RAuMNPs) in a multiplexed LFA for the detection of AFP, CEA, and PSA [189]. These NPs consist of a 150 nm magnetic iron core, initially coated by 20 nm AuNP seeds interspersed with smaller 3 nm AuNPs seeds [189]. The final raspberry-like nanostructure is synthesized when the AuNP seeds are grown on the surface of the iron core via the nucleation reaction of gold chloroauric acid [189]. Nanogaps of 1.4 nm are created between the surface AuNPs of different sizes that generate various hot spots, significantly enhancing Raman signals and giving the RAuMNPs an EF of 2.27 × 10^7^, which was higher than the EF of gold nanospheres, AuNRs, AuSts, and other Fe_3_O_4_@Au magnetic nanoparticles with a smooth gold coating [171–175, 189]. Additionally, the RAuMNPs also function as magnetic beads to separate and enrich the target analyte before using the LFA. This sample enrichment in conjunction with Raman enhancement culminated in the sensitive detection of AFP, CEA, and PSA in bovine serum samples, with low LODs of 1.92 pg/mL, 3.84 pg/mL, and 1.43 pg/mL respectively [189].

Among all the devices listed in Table 4, Shen et al*.*’s approach yielded the lowest LODs measured with spiked serum samples [189]. This method can potentially be applied to other types of biomarkers that require enrichment procedures, such as CTCs. However, this could also present a shortcoming for POC settings, as the additional preparation steps might be cumbersome and time-consuming. In contrast, Gao et al*.* developed a SERS-based LFA for the direct detection of CEA from whole blood, eliminating the need for sample pre-treatment [190]. While not as sensitive as other devices mentioned here (1 ng/mL LOD), it stands out as the device that is closest to satisfying the ASSURED criteria, second only to the urine-based LFA for miRNA [176, 190].

**Table 4:** Paper-based SERS assay for the detection of cancer-derived circulating biomarkers

Sample	Device	SERS substrate	Biomarker	LOD in biological samples^a^	Merits	Demerits	Clinical Samples	Ref.
Serum	LFA	Palladium gold core–shell nanorods (Pd-AuNRs)	TP53, E545K	37.8 aM, 23.1 aM	45 min operation time; results comparable to qRT-PCR; processes up to 8 samples	Large volume of sample needed (150 µL) for testing	30 healthy; 120 laryngeal squamous cell carcinoma samples at different stages	[172]
Serum	LFA	Palladium gold core–shell nanorods (Pd-AuNRs)	miR-106b, miR-196b	43.1 aM, 61.4 aM	Rapid (30 min); results comparable to qRT-PCR	High volume of 100 µL sample required	30 healthy; 120 laryngeal squamous cell carcinoma samples at different stages	[171]
	Filter paper	Au–Ag nanowires (AgNW@AuNPs)	miR-196a	130 aM	Simple operation procedure; results consistent with RT-PCR	2 h operation time; complex manufacturing procedure	8 healthy and 30 lung cancer samples	[191]
Urine	LFA	Gold nanocages	miR-21, miR-196a-5p	3.31 pM, 2.18 pM	30 min operation time; results comparable to qRT-PCR; detecting 2 biomarkers in a single test line reduces fabrication time and reagent use; non-invasive sample collection	Lengthy fabrication of SERS tags	30 healthy and 30 non-small cell lung carcinoma samples	[176]
Serum	VFA	Gold nanostars with Raman reporter encapsulated by silica shell (AuSts@Raman@SiO_2_ NPs)	MUC1, HER2, CEA	(1.00 –2.90) × 10^7^ particles/mL^b^ for MCF-7, SKBR-3, MDA-MB-231, and BT474 exosomes	10 min operation time; no hook effect due to VFA format; good discrimination of protein expression profiles in mixed samples	Modified syringe and removal of conjugate pad for testing might complicate fabrication and user-friendliness; high volume of 75 µL isolated exosomes required; lengthy SERS nanotag fabrication (> 24 h)	5 healthy and 21 breast cancer samples	[177]
Serum	LFA	Gold nanostars with Raman reporter encapsulated by silica shell (AuSts@Raman@SiO_2_ NPs)	HER2, MUC1	3.27 × 10^6^ SKBR-3 exosomes/mL^b^, 4.80 × 10^6^ MCF-7 exosomes/mL^b^	15 min operation time; results comparable to ELISA; multivariate spectral unmixing analysis helps identify exosome subtypes in a mixed sample	High volume of 100 µL isolated exosomes required; requires knowledge of multivariate spectral unmixing	15 healthy; 39 breast cancer samples	[178]
Serum	LFA	Gold nanorods (AuNRs)	AFP	11.7 pg/mL^b^	15 min operation time; only 70 µL sample required; results comparable to CMIA and 100 × more sensitive than ELISA; stable for up to 60 days in storage	LOD was measured with spiked fetal calf serum samples	10 clinical human serum samples	[192]
Serum	LFA	Nano-Ag polydopamine nanospheres (PDA@Ag@Raman NPs)	SCCA, CA125	8.10 pg/mL, 7.37 pg/mL	20 min operation time; only 100 µL sample required; results comparable to ELISA; detecting 2 biomarkers in a single test line reduces fabrication time and reagent use	Lengthy SERS nanotag fabrication (> 40 h for PDA nanospheres)	30 healthy and 120 cervical cancer samples at different stages	[184]
Serum	LFA	Raspberry-like Fe3O4@Au magnetic nanoparticles (RAuMNPs) with nanogaps	PSA, AFP, CEA	1.43 pg/mL^b^, 1.92 pg/mL^b^, 3.84 pg/mL^b^	30 min operation time; high SERS enhancement factor from nanogap hot spots; lower LOD than ELISA; clinical results consistent with CMIA; magnetic NPs could extract other targets such as CTCs	LOD was measured with spiked bovine serum samples; complex synthesis of RAuMNPs might incur higher manufacturing costs	5 hepatocellular carcinoma and 5 prostate cancer samples	[189]
Serum	LFA	Ag/Raman reporter/Au embedded on NC membrane, Au core-Ag shell (Au@Raman @Ag@antibody)	CEA, NSE	39.0 pg/mL^b^, 46.0 pg/mL^b^	15 min operation time; results consistent with ECLIA; 3.2-fold lower LOD than conventional SERS-LFAs due to plasmonic internal standard; detecting 2 biomarkers in a single test line reduces fabrication time and reagent use	LOD was measured with spiked PBS; complex assay assembly; conventional SERS-LFA was made in-house; not comparable with SERS-LFAs from other groups	12 healthy and 12 early-stage lung cancer samples	[188]
Serum	LFA	Graphene oxide/AuNPs (GO/AuNPs) on NC membrane, Au core-Ag shell (Au@Raman @Ag@antibody)	AFP	80.0 pg/mL^b^	20 min operation time; 1.65-fold more reproducible and 3.5-fold lower LOD than conventional SERS-LFAs due to plasmonic internal standard	Unclear if biological samples used for LOD testing; conventional SERS-LFA was made in-house; not tested with cancer patient samples; complex assay assembly; spherical AuNPs have lowest SERS enhancement factor	Unknown number of normal serum samples spiked with PSA	[187]
Plasma	VFA	Raman dye (RD) encoded core–shell (Au@Ag) SERS nanotags	PSA, CEA, AFP	0.370 pg/mL, 0.430 pg/mL, 0.260 pg/mL	7 min operation time; results comparable to ELISA; only 10 µL sample required; detecting of biomarkers in a single dot reduces fabrication time and reagent use; no hook effect	LOD was not tested with biological samples; low number of replicas	5 prostate cancer samples	[183]
Plasma	LFA	Au–Ag hollow nanoparticles (Au–Ag HNPs)	Thrombin, PDGF-BB	4.84 pg/mL, 3.80 pg/mL	30 min operation time; results comparable to ELISA	Plasma isolation and preparation required; clinical samples were not sourced from early-stage cancer patients	30 healthy and 30 prostate cancer samples	[193]
Serum	Filter paper	Au–Ag nanoshuttles; Au nanoflowers embedded on filter paper	SCCA, OPN	8.63 pg/mL, 4.39 pg/mL	Inexpensive assay fabrication; results comparable to ELISA	2 h operation time; lengthy assay fabrication (> 18 h)	30 healthy and 120 cervical cancer samples at different stages	[186]
Serum	Filter paper	Gold nanourchins (GNUs)	Cytochrome c	1.79 pg/mL	Results comparable to ELISA	2 h operation time; lengthy assay fabrication, requires a humid chamber	30 healthy and 30 non-small cell lung cancer samples	[194]
Whole blood^b^	LFA	Gold nanostars with Raman reporter encapsulated by silica shell	CEA	1.00 ng/mL	No sample preparation required, enabling immediate use in POC settings	Lower plasma yield compared to centrifugation (30 vs 50%); lower sensitivity than ELISA	No	[190]

^a^Without pre-treatment

^b^isolated from biological samples or tested in spiked biological samples

All in all, the integration of SERS into paper-based assays has significantly increased sensitivity and enabled the detection of scarce biomarkers important for early disease diagnosis and monitoring. Table 4 has summarized the paper-based SERS assays for the detection of ctNAs, circulating tumor-derived sEVs and related proteins that were developed in the last 5 years. Paper-based SERS assays are relatively unexplored in comparison with other types of SERS-based detection assays, particularly for the detection of CTCs, highlighting a significant gap in the current research. Despite the enhanced sensitivity, challenges in terms of portability and user-friendliness persist due to the reliance on external equipment to measure Raman signals. This makes it difficult for assays to meet the stringent ASSURED criteria for POC tests outlined by WHO. Nevertheless, advances such as new shapes of nanoparticles with more hot spots for enhancing Raman signals, the integration of a plasmonic internal standard for reducing background signals, and utilization of multivariate spectral unmixing for distinguishing between complex samples are crucial for the continued improvement of paper-based assay technologies that have the potential to revolutionize disease detection and monitoring.

## Conclusions, perspectives and outlook

In conclusion, liquid biopsy based cancer diagnosis benefits from a diverse range of SERS biosensor platforms, including label-free SERS assay, bead-based sensors, microfluidic device systems, and paper-based assays, each demonstrating unique capabilities in enhancing the sensitivity and specificity of circulating cancer biomarker detection: *i)* label-free SERS assays explore the elimination of exogenous SERS labels, simplifying assay procedures and facilitating real-time detection of circulating biomarkers, thereby accelerating diagnostic processes, *ii*) bead-based SERS biosensors leverage functionalized beads to capture and analyze diverse circulating biomarkers, demonstrating potential for multiplexed detection and precise characterization; *iii)* microfluidic device-based biosensors represent a frontier in liquid biopsy, enabling precise manipulation of small sample volumes, high-throughput analysis, and integration with SERS technology for enhanced sensitivity and rapid biomarker detection; *iv)* paper-based SERS biosensors offer portable and cost-effective diagnostic solutions, widening access to liquid biopsy technologies and enabling point-of-care applications. Therefore, this comprehensive review illuminates the transformative impact of SERS-based biosensors in liquid biopsy, empowering accurate and non-invasive detection of circulating biomarkers for improved cancer diagnosis and treatment management.

The continued evolution of SERS biosensors holds immense potential in several key areas, including *i)* The paradigm of label-free SERS assays is anticipated to witness extensive exploration, driven by the quest for simplification and real-time detection. Innovations in plasmonic nanostructures paves the way for highly sensitive label-free SERS assays, minimizing complexities in sample preparation and expediting diagnostic workflows; *ii)* The advancement of beads-based SERS biosensors, with their capacity for multiplexed analysis and improved capture efficiency, presents an opportunity for simultaneous profiling of multiple biomarkers, enabling a more comprehensive understanding of tumor heterogeneity and aiding in personalized treatment strategies; *iii)* Microfluidic device-based biosensors are anticipated to undergo refinements in design and functionality, enabling seamless integration with SERS technology. The future integration of microfluidic systems with advanced SERS detection promises enhanced throughput, precise manipulation of minute sample volumes, and real-time analysis, catalyzing the translation of liquid biopsy into routine clinical practice; *iv)* The trend of paper-based SERS biosensor is poised towards further miniaturization, cost-effectiveness, and integration with portable devices, facilitating decentralized cancer diagnostics. This evolution could revolutionize resource-limited settings and empower healthcare providers with rapid, on-site diagnostic capabilities, thereby addressing accessibility gaps in cancer care.

Despite these promising prospects, several challenges persist in the field of SERS biosensors for liquid biopsy. Standardization of protocols, validation of assays, and harmonization of data analysis remain pivotal for ensuring reproducibility and reliability across different platforms. Additionally, addressing concerns regarding cost, scalability, and regulatory consideration will be imperative for their widespread adoption in clinical settings.

In summary, the convergence of SERS biosensors with liquid biopsy represents a new era in cancer diagnostics, offering non-invasive, sensitive, and multiplexed detection of circulating biomarkers. The future trend involves concerted efforts towards technological refinements, addressing challenges, and fostering collaborations between academia, industry, and regulatory bodies to propel SERS-based liquid biopsy into routine clinical practice, ultimately revolutionizing cancer diagnosis and patient care.

## Data Availability

This review article synthesizes and analyses data from previously published studies. All data referenced in this review are available from the original sources, which are cited in the references section.
